# Construction of the systemic anticancer immune environment in tumour-bearing humanized mouse by using liposome-encapsulated anti-programmed death ligand 1 antibody-conjugated progesterone

**DOI:** 10.3389/fimmu.2023.1173728

**Published:** 2023-07-10

**Authors:** Yoshie Kametani, Ryoji Ito, Shino Ohshima, Yoshiyuki Manabe, Yusuke Ohno, Tomoka Shimizu, Soga Yamada, Nagi Katano, Daiki Kirigaya, Keita Ito, Takuya Matsumoto, Banri Tsuda, Hirofumi Kashiwagi, Yumiko Goto, Atsushi Yasuda, Masatoshi Maeki, Manabu Tokeshi, Toshiro Seki, Koichi Fukase, Mikio Mikami, Kiyoshi Ando, Hitoshi Ishimoto, Takashi Shiina

**Affiliations:** ^1^ Department of Molecular Life Science, Division of Basic Medical Science, Tokai University School of Medicine, Isehara, Japan; ^2^ Institute of Advanced Biosciences, Tokai University, Hiratsuka, Kanagawa, Japan; ^3^ Human Disease Model Laboratory, Department of Applied Research for Laboratory Animals, Central Institute for Experimental Animals, Kawasaki, Japan; ^4^ Department of Chemistry, Graduate School of Science, Osaka University, Osaka, Japan; ^5^ Forefront Research Center, Osaka University, Osaka, Japan; ^6^ Department of Palliative Medicine, Tokai University School of Medicine, Isehara, Japan; ^7^ Department of Obstetrics and Gynecology, Tokai University School of Medicine, Isehara, Japan; ^8^ Department of Internal Medicine, Division of Nephrology, Endocrinology, and Metabolism, Tokai University School of Medicine, Isehara, Japan; ^9^ Faculty of Engineering, Hokkaido University, Sapporo, Japan; ^10^ Department of Hematology and Oncology, Tokai University School of Medicine, Isehara, Japan; ^11^ Department of Hematology and Oncology, Research Institute for Radiation Biology and Medicine, Hiroshima University, Hiroshima, Japan

**Keywords:** breast cancer, immune environment, liposome, progesterone, programmed death ligand 1, humanized mouse

## Abstract

Immune checkpoint inhibitors highlight the importance of anticancer immunity. However, their clinical utility and safety are limited by the low response rates and adverse effects. We focused on progesterone (P4), a hormone produced by the placenta during pregnancy, because it has multiple biological activities related to anticancer and immune regulation effects. P4 has a reversible immune regulatory function distinct from that of the stress hormone cortisol, which may drive irreversible immune suppression that promotes T cell exhaustion and apoptosis in patients with cancer. Because the anticancer effect of P4 is induced at higher than physiological concentrations, we aimed to develop a new anticancer drug by encapsulating P4 in liposomes. In this study, we prepared liposome-encapsulated anti-programmed death ligand 1 (PD-L1) antibody-conjugated P4 (Lipo-anti-PD-L1-P4) and evaluated the effects on the growth of MDA-MB-231 cells, a PD-L1-expressing triple-negative breast cancer cell line, *in vitro* and in NOG-hIL-4-Tg mice transplanted with human peripheral blood mononuclear cells (humanized mice). Lipo-anti-PD-L1-P4 at physiological concentrations reduced T cell exhaustion and proliferation of MDA-MB-231 *in vitro*. Humanized mice bearing MDA-MB-231 cells expressing PD-L1 showed suppressed tumor growth and peripheral tissue inflammation. The proportion of B cells and CD4+ T cells decreased, whereas the proportion of CD8+ T cells increased in Lipo-anti-PD-L1-P4-administrated mice spleens and tumor-infiltrated lymphocytes. Our results suggested that Lipo-anti-PD-L1-P4 establishes a systemic anticancer immune environment with minimal toxicity. Thus, the use of P4 as an anticancer drug may represent a new strategy for cancer treatment.

## 1 Introduction

Immune checkpoint blockade is a powerful tool in cancer treatment. However, the overall treatment response rate is low and patients experience acute and chronic immune-related adverse events, suggesting that systemic anticancer immune responses are impaired by the establishment of a local tumor microenvironment (1, 2). Cancers of different origins have developed similar strategies for evading the immune system. For example, cancer cells frequently escape immune surveillance by downregulating the major histocompatibility complex expression and/or expressing programmed death ligand 1 (PD-L1) (3, 4). Cancer cells share these characteristics and gene expression patterns with placental trophoblasts (5). However, trophoblast invasion of the endometrium and decidua during pregnancy is highly regulated; the invasion stops in the myometrium to prevent dispersion into the maternal circulation (6, 7). After 10 months of gestation, trophoblasts are excreted or undergo apoptosis. The immune system of pregnant women is active in order to protect against infectious diseases; this was evident by the statistically insignificant difference between the death rate of pregnant women with coronavirus disease 2019 and that of non-pregnant women (8, 9). Moreover, pregnancy does not promote cancer progression (10).

We tried to construct an anticancer immune environment by using molecules involved in the immune system during pregnancy. We focused on progesterone (P4), which is produced in the placenta during pregnancy (11), because it is a multiple-target transcription factor and has various biological activities, such as regulation of the maternal–fetal tolerance (12) and anticancer effects (13, 14). P4 inhibits epithelial-to-mesenchymal transition (15), and its derivative induces the transition from endoplasmic reticulum stress to apoptosis (16). P4 also affects mitochondrial processes involved in apoptosis induction (17). Resting T cells survive and retain competency in the presence of P4 but die when activated by a high concentration of it (18). We also found that P4 treated cells expanded CD8 T cells extensively in humanized mice (18). Therefore, if a high amount of P4 is administered, both cancer cells and activated T cells that induce immune-related adverse events (irAEs) will die and naive T cells and resting memory T cells will survive; thus, cancer growth and irAEs might be reduced and naïve T cells might activate after the treatment. Especially, cytotoxic T cells might proliferate to induce high anticancer effects.

Glucocorticoids induce T cell exhaustion irrespective of activation state (18). Glucocorticoids suppress T cell effector functions by secreting IL-10 following the irreversible induction of T cell exhaustion (19). The use of glucocorticoids in patients with cancer which show irAEs may promote cancer progression owing to the immunosuppressive function of glucocorticoids (20). Thus, P4 may be a suitable alternative to glucocorticoids. However, the concentration of P4 required to induce antitumor effects and immune regulation is higher than its normal physiological levels.

Therefore, effective delivery is essential to achieve the dual benefits of P4. Large quantities of exosomes are secreted into placental villi (21), the contents of which reflect those of the parent cells (22). As syncytiotrophoblasts produce large amounts of P4, exosomes may contain high concentrations of hydrophobic P4 in their plasma and/or endoplasmic reticulum membranes. To mimic intrauterine exosomes, we encapsulated P4 in liposomes, which have been extensively investigated and whose toxicity and safety have been evaluated. To specifically target cancerous cells, P4 has been conjugated to the anti-PD-L1 monoclonal antibody atezolizumab (23). To study the effects of Lipo-anti-PD-L1-P4, it is insufficient to use conventional experimental animals, because the immune system is specific to each species (24). Therefore, we developed a humanized mouse model using NOD/SCID/γcnull (NOG) mice (25); it can be used to evaluate patient immunity—especially humoral immunity (26). We developed a second-generation immunodeficient mouse model (NOG-hIL-4-Tg) that expressed human IL-4 (27). These mice did not develop graft-versus-host disease after the transplantation of human peripheral blood mononuclear cells (PBMC). Mature human B cells can be engrafted into NOG-hIL-4-Tg mice, suggesting that the human immune environment can be recreated. We found that the immunity of healthy donors and patients with breast cancer could be compared by transplanting PBMCs into NOG-hIL-4-Tg mice (28). In this study, we evaluated the effects of Lipo-anti-PD-L1-P4 by analyzing the kinetic profile and localization of lymphocytes *in vitro* and *in vivo* using a humanized mouse model.

## 2 Materials and methods

### 2.1 Ethical approval

This study was conducted in accordance with the guidelines of the Declaration of Helsinki and Japanese federal regulations required for the privacy protection of human participants. The study protocol was approved by Tokai University (12R-002/20R211/21R277) and the Central Institute for Experimental Animals (08-01) Human Research Committees. Written informed consent was obtained from all participants. All animal procedures were conducted in accordance with the Guidelines for the Care and Use of Laboratory Animals and were approved by the Animal Care Committees of Tokai University School of Medicine (185016/191073/202049/213047/224039) and the Central Institute for Experimental Animals (20045). The study was conducted in compliance with the ARRIVE guidelines. As we created the healthy donor’s immune environment in multiple mice, the experimental design did not need blinding.

### 2.2 Cell lines

The human triple-negative breast adenocarcinoma (MDA-MB-231) cell line was purchased from the European Collection of Authenticated Cell Cultures, cultured in L15 medium supplemented with 15% fetal calf serum (FCS) (Sigma-Aldrich, St. Louis, MO, USA), and maintained at 37°C without CO_2_ equilibration. The myeloma (Karpas 707H) cell line was kindly gifted by Dr. Karpus from Cambridge University. Human placental choriocarcinoma (JEG-3 and BeWo) and embryonic kidney (HEK-293) cells were purchased from the American Type Collection Center (ATCC) (CLS Cat# 300192/p777_HEK293, RRID:CVCL_0045) and stored at the Tokai University School of Medicine. JEG-3 cells were cultured in low-glucose DMEM supplemented with 10% FCS and maintained at 37°C under 5% CO_2_. BeWo cells were cultured in Ham’s F12 medium supplemented with 10% FCS and maintained at 37°C under 5% CO_2_. HEK-293 cells were cultured in high-glucose DMEM supplemented with 10% FCS and maintained at 37°C in 5% CO_2_. Karpas 707H cells were cultured in RPMI-1640 medium (Nissui Co. Ltd., Tokyo, Japan) supplemented with 10% FCS and maintained at 37°C under 5% CO_2_.

### 2.3 Human PBMCs

Peripheral blood samples (approximately 30 mL) from 42 healthy donors without a history of malignancy (23 males and 19 female individuals; age, 21–64 years) were collected in Vacutainer ACD tubes containing heparin (Becton Dickinson, Franklin Lakes, NJ, USA). The samples were immediately placed in 10 mL of Ficoll-Paque PLUS (Cytiva, London, UK). PBMCs were isolated under density gradient centrifugation (500 ×*g* for 30 min at 20°C) and washed with phosphate-buffered saline (PBS) (300 ×*g* for 5 min at 4°C).

### 2.4 *In vitro* culture of PBMCs

The PBMCs (1 × 10^6^/mL) were stimulated with 1 μg/mL of toxic shock syndrome toxin 1 (TSST-1;Toxin Technology Inc., Sarasota, FL, USA) in RPMI-1640 medium supplemented with 10% fetal FCS and antibiotics (streptomycin, 0.1 mg/mL; penicillin, 100 U/mL [Meiji Seika, Tokyo, Japan]) in the presence or absence of different concentrations of P4 (Sigma-Aldrich), cortisol (Sigma-Aldrich), atezolizumab (Chugai Pharmaceutical Co. Ltd., Tokyo, Japan), or liposome-encapsulated atezolizumab-conjugated steroids, and cultured for 72 h at 37°C under 5% CO_2_. The cells were harvested and washed with PBS, and 5 × 10^5^ cells/tube were used for flow cytometry.

### 2.5 Flow cytometry

Fluorochrome-conjugated anti-human monoclonal antibodies were used to identify human immune cells. Cells were incubated with appropriate dilutions of fluorescently labeled mouse anti-human monoclonal antibodies (**Table S1**) for 15 min at 4°C, washed with PBS containing 1% (w/v) bovine serum albumin (Sigma-Aldrich), and analyzed using FACS Fortessa or Verse flow cytometer (BD Biosciences, Franklin Lakes, NJ, USA). For each analysis, a live gate with white blood cells or lymphocytes was used based on human CD45 expression. The data were analyzed using FlowJo, PRID:SCR_008520 (BD Biosciences).

The culture supernatants were collected. Debris was removed by centrifugation (300 ×*g* 5 *min* at 4°C). Cytokine quantification was performed using LEGENDplex v8.0 (BioLegend, San Diego, CA, USA). Briefly, 25 µL of the supernatant was mixed with 25 µL of capture beads and incubated for 2 h at 25 °C. The beads were washed, mixed with detection antibodies, and incubated for 1 h at 25 °C. Streptavidin–phycoerythrin was added, and the mixture was incubated for 30 min at 25 °C. The beads were washed and analyzed using a FACSVerse flow cytometer (BD Biosciences). Data (pg/mL) were analyzed using LEGENDplex v8.0.

### 2.6 NOG-hIL-4-Tg mice

P4- or cortisol-treated human PBMCs were transplanted into NOG-hIL-4-Tg (NOD.Cg-Prkdc*^scid^* Il2rg*^tm1Sug^*/Tg(CMV-IL4)/Jic) mice, which were maintained under specific pathogen-free conditions at the animal facilities of the Tokai University School of Medicine and the Central Institute for Experimental Animals. To determine the effector function of P4- or cortisol-treated human PBMCs, T and B cells were evaluated because human PBMCs can engraft in NOG-hIL-4-Tg mice without causing graft-versus-host disease (27). Offspring expressing human IL-4 were identified as previously described (27). DNA was extracted from the ear tissue collected at the time of genotyping. Human IL-4 protein level was measured using the Human IL-4 ELISA Set BD OptEIA Kit (BD Biosciences).

### 2.7 Lipo-anti-PD-L1-P4

First, the following solutions were prepared: lipid solution (1,2-dioleoyl-*sn*-glycero-3-phosphocholine [35.8 mg (45.5 μmol)] [COATSOME MC-8181; NOF Corp., Tokyo, Japan] and *N*-[(3-maleimide-1-oxopropyl)aminopropyl polyethyleneglycol-carbamyl] distearoylphosphatidyl-ethanolamine [11.7 mg (4.0 μmol)] [SUNBRIGHT DSPE-020MA; NOF Corp.]) and P4 (9.56 mg [30.4 μmol]) (160-24511; FUJIFILM Wako Pure Chemical Industries Ltd., Osaka, Japan) in ethanol (5.0 mL). The lipid solution and 25 mM acetate buffer (pH 4.0) were injected into a microfluidic device (iLiNP) at flow rates of 50 and 150 μL/min, respectively, using microsyringes and microsyringe pumps (70-2208; Harvard Apparatus, Holliston, MA, USA) (29). The mixed solution from the outlet was collected in a microtube (18 ml) and dialyzed against PBS for 12 h at 4°C using Spectra/Por 4 Dialysis Membrane Standard RC Tubing (12−14 kDa cutoff) (08-667B; Spectrum Laboratories Inc., San Francisco, CA, USA). The solution containing P4 liposomes (15 mL) was concentrated to 4.5 mL using the Amicon Ultra-15 Centrifugal Filter Device (100 kDa cutoff) (UFC910024; Merck Millipore, Burlington, MA, USA) at 1,000 ×*g* for 70 min at 4°C. The size and ζ-potential of P4 liposomes were measured using a ZetaSizer Ultra (Malvern Instruments, Worcestershire, UK). The concentration of P4 incorporated into liposomes was quantified using high-performance liquid chromatography (column: COSMOSIL 5C18-AR-300 2.0 × 150 mm [37991-61; Nacalai Tesque, Kyoto, Japan]; eluent: linear gradient 2%→98% acetonitrile aqueous solution containing 0.1% trifluoroacetic acid for 48 min, followed by isocratic elution of 98% acetonitrile aqueous solution containing 0.1% trifluoroacetic acid for 7 min; flow rate: 0.2 mL/min; detection: 220 nm UV) (Shimadzu Prominence UFLC; Shimadzu Corp., Kyoto, Japan). This quantification revealed that out of the 8.6 mg of P4 introduced to the iLiNP device, 0.71 mg of it was encapsulated in liposomes (8%), and all experiments were performed based on this quantification. Given that all its constituent lipids (DOPC and DSPE-PEG-MAL) are incorporated into the liposome, the P4 content in the liposomes was calculated to be 1.5% (w/w).

P4 leakage test: P4-loaded liposomes (100 µL) were placed in a dialysis membrane (14 kDa cutoff, 046-30911; FUJIFILM Wako Pure Chemical Industries Ltd., Osaka, Japan) and dialyzed with more than 1,000-flod PBS at 37°C. The decrease in P4 concentration in the dialysis tube was monitored over time. These experiments revealed that P4 was gradually released over a period of approximately 12 h (**Figure S1A**).

Half-antibody-conjugated P4 liposomes were prepared as follows: 2-mercaptoethanol (8.8 mL [120 μmol]; final concentration: 50 mM) (135-07522; FUJIFILM Wako Pure Chemical Industries Ltd.) and ethylenediaminetetraacetic acid (3.5 mg [12 μmol]; final concentration: 5 mM) (15105-35; Nacalai Tesque) were added to atezolizumab in PBS (2.4 mL [2.0 mg/mL]). After incubation at 37 °C for 1.5 h, the solution was centrifuged using the Amicon Ultra-15 Centrifugal Filter Device (10 kDa cutoff) (UFC901024; Merck Millipore) at 2,580 ×*g* for 30 min at 4°C. PBS (14 mL) was added to the high molecular weight fraction and centrifuged again. This procedure was repeated thrice. Protein concentration was adjusted to 1 mg/mL and measured using a Nanodrop Lite spectrophotometer (ND-LITE-PR; Thermo Fisher Scientific, Waltham, MA, USA). Half-antibody (1 mg/mL) and P4 liposome (78.5 μg/mL) solutions were mixed in a 1:1 (v/v) ratio and shaken for 2 h at 25°C to obtain half-antibody-conjugated P4 liposomes (Lipo-anti-PD-L1-P4).

### 2.8 Transplantation and treatment of NOG-hIL-4-Tg mice

Human PBMCs (5 × 10^6^ cells) were injected intravenously into 8-week-old NOG-hIL-4-Tg mice. Atezolizumab (250 μg/head), Lipo-anti-PD-L1-P4 (250 μg atezolizumab was contained in the administered liposome/head), and empty liposomes (250 μg atezolizumab was contained in the administered liposome/head) were administered intraperitoneally every five days for a total of six times. As a negative control, an equal volume of PBS was injected into NOG-hIL-4-Tg mice transplanted with human PBMCs (hu-PBL hIL-4 NOG mice). The mice were sacrificed 28 **d** after the PBMC transplantation.

For the tumor-bearing humanized mouse model, 5 × 10^6^ MDA-MB-231 cells were transplanted subcutaneously into the flanks of 7-to 9-week-old NOG-hIL-4-Tg mice. After two weeks, 5 × 10^6^ PBMCs were transplanted intravenously. Atezolizumab, Lipo-anti-PD-L1-P4, empty liposomes, and PBS were administered every five days intraperitoneally. Solid tumor diameters were measured every alternate day using a micrometer caliper. The tumor volume was calculated as {day X long diameter (mm) × short diameter (mm) × short diameter (mm) - day-0 long diameter (mm) × short diameter (mm) × short diameter (mm)}/2.

### 2.9 Immunohistochemistry

NOG-hIL-4-Tg mouse spleen, lung, liver, and tumor tissues were fixed in Mildform (FUJIFILM Wako Pure Chemical Industries, Ltd.) and embedded in paraffin. The paraffin blocks were micro-sectioned and deparaffinized. Post-fixed tissue sections were stained with hematoxylin and eosin.

For immunohistochemistry, the tissues were fixed in formalin, washed, and mounted onto glass slides. Endogenous peroxidase activity was blocked with 1% goat serum for 10 min at 25°C. The sections were blocked with goat serum for 30 min, washed, and incubated with primary monoclonal antibodies (**Table S1**). Subsequent incubation with peroxidase-labeled anti-mouse Ig antibody (Histofine Simple Stain MAX-PO; Nichirei Biosciences INC, Tokyo, Japan) was performed according to the manufacturer’s protocol.

### 2.10 Quantification of cytokine secretion

NOG-mouse spleen cells, previously stored at -80°C, were stained with anti-human CD4 and CD8 antibodies, and CD4 and CD8 T cells were sorted using the FACSAria Cell sorter (BD Biosciences). For the CD3+ cells, the Pan T Cell Isolation Kit (Miltenyi Biotec B.V. & CO. KG, Germany) was used for T cell sorting. Briefly, the cells were washed and incubated with the Pan T cell biotin-antibody cocktail at 4°C for 5 min. Afterwards the mixture was washed with wash buffer and 20 μL of Pan T cell micro bead cocktail was added and then incubated at 4°C for 10 min. The T cells were sorted using the Automacs system (program: depletion; Miltenyi Biotec). The purified cells (1x10^5^/well) were stimulated with Dynabeads™ Human T-Activator CD3/CD28 (Thermo Fisher Scientific) at 37°C, 5% CO_2_ for 72 h. Supernatants of the cultured cells were submitted for cytokine quantitation using the bead-based multiplex LEGENDplex (BioLegend) according to the manufacturer’s instructions. Briefly, the supernatant was mixed with capture beads and incubated for 2 h at 25°C. The beads were then washed and incubated with detection antibodies for 1 h at 25°C. Subsequently, streptavidin-phycoerythrin was added, and the mixture was incubated for 30 min at 25°C. Finally, the beads were washed and analyzed using FCM as mentioned above. Then the IL-5, IL-13, IL-2, IL-6, IL-9, IL-10, IFN- γ, TNF- α, IL-17A, IL-17F, IL-4, and IL-22 cytokines were quantified. The analysis was performed using the BD FACSVerse™ Flow Cytometer (BD Biosciences). The data were analyzed in pg/mL using LEGENDPlex™ V8.0 (BioLegend).

### 2.11 Statistical Analysis

Data are presented as mean ± standard deviation. After the normality testing, significant differences between groups were determined using a one-way analysis of variance or two-sided Student’s *t*-test for *in vitro* analyses. Regarding the mice data, we conducted a Mann-Whitney U test and evaluated the statistical significance. All statistical analyses were performed in Microsoft Excel, RRID:SCR_016137 (Microsoft Corp., Redmond, WA, USA).

## 3 Results

### 3.1 P4 suppresses T Cell activation and tumor growth

**Figure 1:**
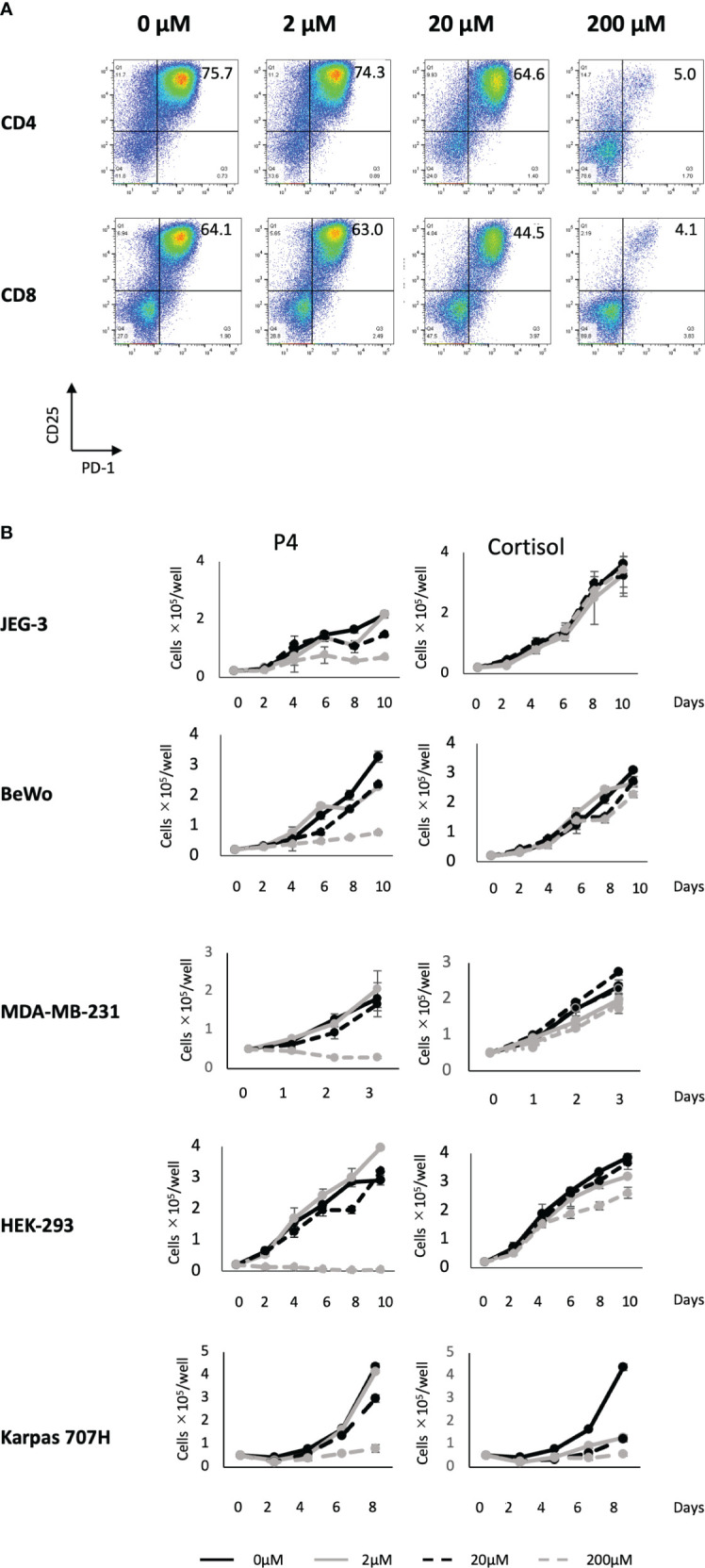
Effect of P4 on human T cells and cancer cell lines. **(A)** Peripheral blood mononuclear cells (PBMCs) were stimulated with toxic shock syndrome toxin 1 in the presence of 0, 2, 20, and 200 μM of P4. T cell activation was analyzed using flow cytometry. Upper panels: CD4+ T cells; lower panels: CD8+ T cells. The levels of activated CD25+/PD-1+ T cells are shown. **(B)** JEG-3, BeWo, MDA-MB-231, HEK-293, and Karpas 707H cells cultured with P4 (left panels) or cortisol (right panels). Solid black lines: 0 μM; broken black lines: 2 μM; solid gray lines: 20 μM; broken gray lines: 200 μM. Data are presented as the mean ± standard deviation of three independent experiments. P4, progesterone.

We previously reported that a high concentration (200 μM) of P4 transiently suppresses T-cell activation (18). This was confirmed by the results presented in **Figure 1A** using the gating strategy shown in **Figure S2**. We activated PBMCs with toxic shock syndrome toxin 1 (TSST-1; a superantigen molecule that can activate non-specific T cells via antigen-presenting cells (APCs)) in the presence (2–200 μM) and absence (0 μM) of P4. T cells expressed the early activation marker CD25 and late activation marker PD-1 after 3 d in the absence of P4. However, the expression of both activation markers was suppressed in the presence of 20–200 μM P4 on both CD4 and CD8 T cells. We also examined whether P4 exerted anticancer effects on cancer cells as previously reported (13, 14). The growth of JEG-3, BeWo, MDA-MB-231, and HEK-293 cells was severely suppressed in the presence of 200 μM P4, whereas no suppression was observed in the presence of cortisol, an immunosuppressive steroid drug. The presence of 20 μM P4 did not significantly suppress the growth of Karpas 707H cells compared with cortisol. However, this suppression was comparable to that of cortisol in the presence of 200 μM P4 (**Figure 1B**).

These results suggest that a high concentration (200 μM) of P4 suppresses T cell activation and tumor growth, especially in adenocarcinomas.

### 3.2 P4 and atezolizumab decrease the expression of immune checkpoint molecules

**Figure 2:**
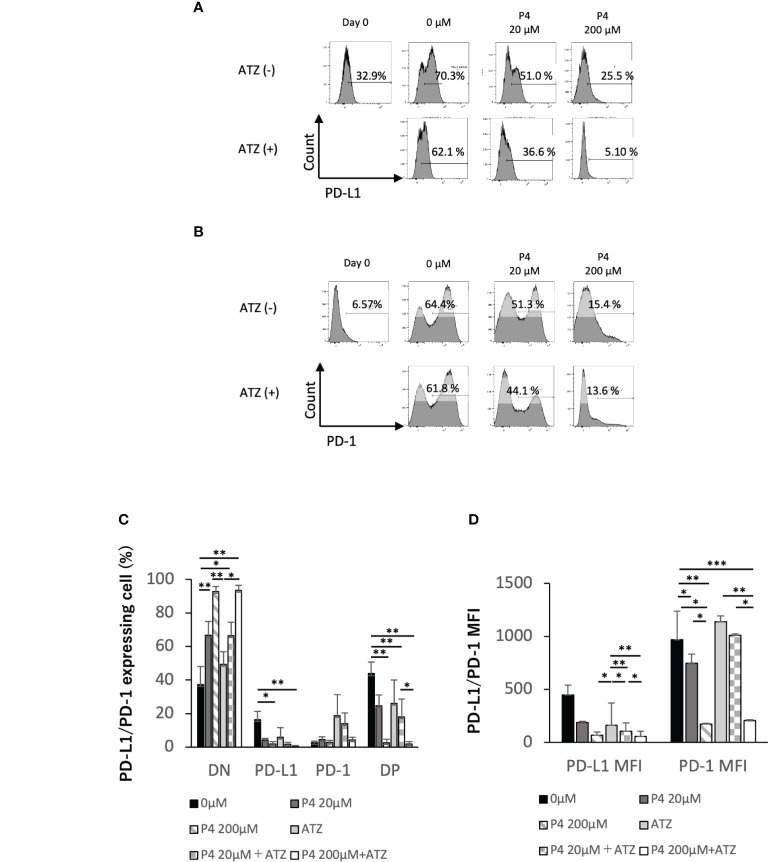
Effect of atezolizumab on the expression of immune checkpoint molecules. **(A)** PD-L1 and **(B)** PD-1 expression on T cells. P4 (0–200 μM) was added to human PBMCs and stimulated with TSST-1 for 72 h. PD-L1 expression in CD3-gated cells are shown as histograms. The culture with/without atezolizumab is shown as [ATZ(+)] or [ATZ (–)]. “Day 0” means the PBMCs before culture and “0μM” means the TSST-1 treatment without P4. Percentages shown in each panel of T cells are the PD-L1(A)- and PD-1(B)-positive cell ratios. Numbers shown in each panel of ATZ, atezolizumab; P4, progesterone; PD-1, programmed death 1; PD-L1, programmed death ligand 1. **(C)** PD-L1/PD-1 expressing cells (%). **(D)** MFI of PD-L1 or PD-1 in the CD3+ cells. Data are presented as mean ± standard deviation (S.D.) **P < 0.01 (one-way analysis of variance or Student’s *t*-test) [*n* = 5].

T cell activation was suppressed when human T cells were stimulated with a high concentration (200 μM) of P4. However, T and B cells may express immune checkpoint molecules, such as programmed death 1 (PD-1) receptor and PD-L1, promoting T cell exhaustion. Because immune checkpoint antibodies suppress T cell exhaustion by blocking the interaction between PD-1 and PD-L1, we hypothesized that the co-administration of P4 and atezolizumab (an anti-PD-L1 monoclonal antibody) may preserve the quiescent state of T cells by preventing T cell exhaustion. Therefore, we activated PBMCs with TSST-1 in the presence (20 and 200 μM) and absence (0 μM) of P4. P4 decreased PD-1 and PD-L1 expression in T cells (**Figures 2A–D**). In the presence of atezolizumab and in the absence of P4, PD-L1 and PD-1 single-positive (SP) cells proportion tended to decrease and increase, respectively, compared to that of the control. Double-positive (DP) cells showed intermediate patterns. Co-administration of P4 and atezolizumab further suppressed PD-1 and PD-L1 expressing T cell proportion, although effective suppression required the administration of P4 at a high concentration (200 μM) of P4. The mean fluorescence intensity (MFI) of PD-1 and PD-L1 expressing cells showed a similar tendency. These results suggest that the co-administration of P4 and atezolizumab is effective in preserving the quiescent state of T cells.

### 3.3 Liposome encapsulation enhances the inhibitory effect of P4

**Figure 3:**
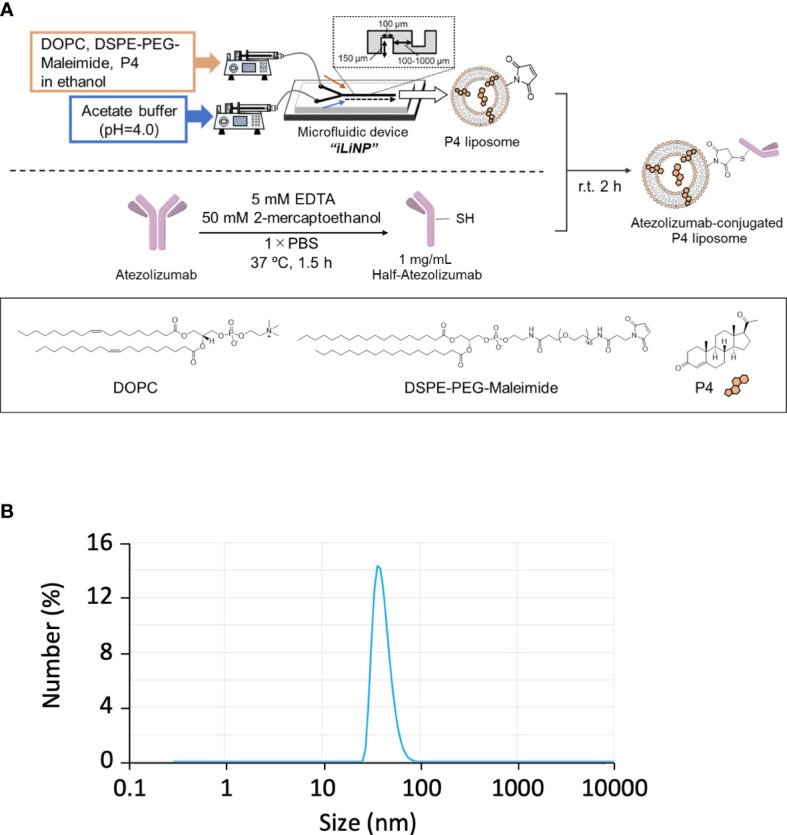
Preparation of Lipo-anti-PD-L1-P4. **(A)** Protocol for the preparation of Lipo-anti-PD-L1-P4. **(B)** Representative result of liposome size distribution. Average size: 44 nm; polydispersity index: 0.22; ζ-potential: –20.1 kV; P4 concentration: 78.5 μg/mL. **(C)** P4 leakage test. DOPC, 1,2-dioleoyl-*sn*-glycero-3-phosphocholine; DSPE-PEG-Maleimide, *N*-[(3-maleimide-1-oxopropyl)aminopropyl polyethyleneglycol-carbamyl] distearoylphosphatidyl-ethanolamine; EDTA, ethylenediaminetetraacetic acid; Lipo-anti-PD-L1-P4, liposome-encapsulated anti-programmed death ligand 1 antibody-conjugated P4; P4, progesterone; PBS, phosphate-buffered saline; r.t., room temperature.

In pregnant women, P4 is mainly synthesized by placental syncytiotrophoblasts. Placental intervillous blood contains approximately 10-fold more P4 than the peripheral blood, with a maximum concentration of approximately 20 μM. Even if P4 is proven to have an immune regulatory effect, the effective dose is too high for clinical administration because it is 10-fold higher than its normal physiological concentration. As syncytiotrophoblasts secrete large amounts of exosomes, we attempted to encapsulate P4 in liposomes (**Figure 3A**) for targeted delivery to cells. As shown in **Figure 3B**, the liposome diameter was <100 nm. The liposome size remained stable for more than one month if maintained at 4°C (**Figure S1B**).

**Figure 4:**
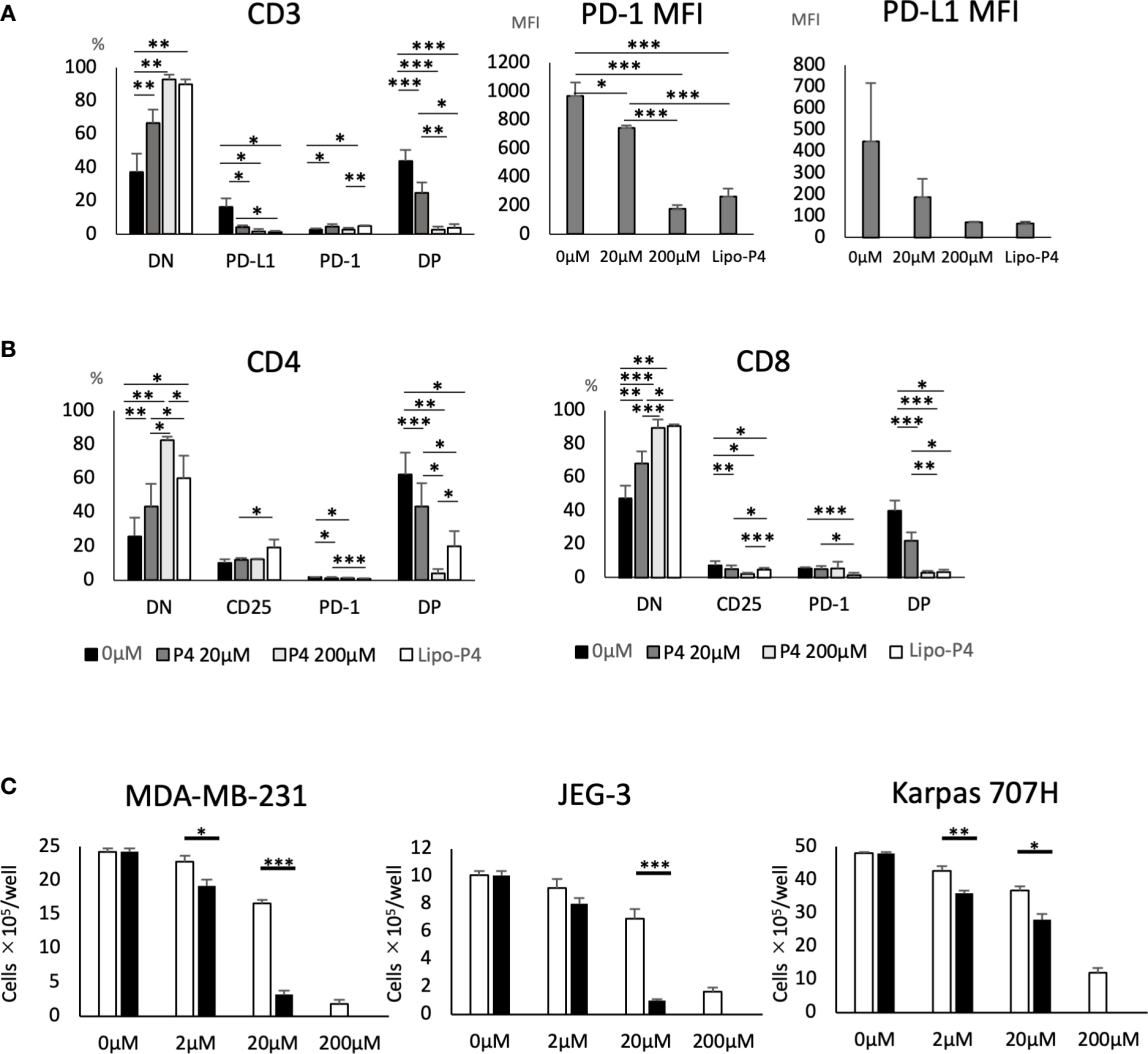
Effect of Lipo-anti-PD-L1-P4 on cell proliferation. PD-1 and PD-L1 expression on T cells **(A, B)** and **(C)** cancer cells. **(A)** Left panel: Percentage of PD-1 and PD-L1 expressing cells in CD3+ T cells. Right panels: PD-1 and PD-L1 mean fluorescence intensities of CD3+ T cells. **(B)** Left panel: percentage of PD-1 and PD-L1 expressing cells in CD4+ T cells; right panel: percentage of PD-1 and PD-L1 expressing cells in CD8+ T cells. **(C)** MDA-MB-231, JEG-3, and Karpas 707H cells were cultured in the presence or absence of P4 (open bars) or Lipo-anti-PD-L1-P4 (solid bars). Vertical lines represent the cell number after 3 d of culture. *P < 0.05; **P < 0.01; ***P < 0.001 (one-way analysis of variance or two-sided Student’s *t*-test). Data are presented as the mean ± standard deviation of four independent experiments. DN, double-negative; DP, double-positive; Lipo-anti-PD-L1-P4 (Lipo-P4), liposome-encapsulated anti-programmed death ligand 1 antibody-conjugated P4; MFI, mean fluorescence intensity; PD-1, programmed death 1; PD-L1, programmed death ligand 1.

Human PBMCs were cultured in the presence of Lipo-anti-PD-L1-P4. Lipo-anti-PD-L1-P4 containing 20 μM P4 significantly suppressed T cell activation, especially in PD-1+/PD-L1+ T cells (**Figure 4A**). The MFI was reduced (**Figure 4A**) and the levels of activated CD25+/PD-1+ T cells in the presence of Lipo-anti-PD-L1-P4 were similar to those of CD8+ T cells in the presence of P4 10-fold higher than the normal levels (**Figure 4B**), suggesting that the construct enhanced the inhibitory effect of P4 on T cells. This construct enabled P4 to regulate the immune system at physiological concentrations.

When JEG-3 and MDA-MB-231 cells, both of which express PD-L1, were cultured *in vitro*, Lipo-anti-PD-L1-P4 containing 20 μM P4 suppressed cell proliferation to the same extent as 200 μM P4. In Karpas 707H cells that did not express PD-L1, Lipo-anti-PD-L1-P4 suppressed cell proliferation to a lesser extent (**Figure 4C**). These results suggest that Lipo-anti-PD-L1-P4 effectively suppresses T cell activation and the growth of PD-L1-expressing cancer cells at physiological concentrations of P4.

### 3.4 Establishment of a tumor-bearing humanized mouse model

**Figure 5:**
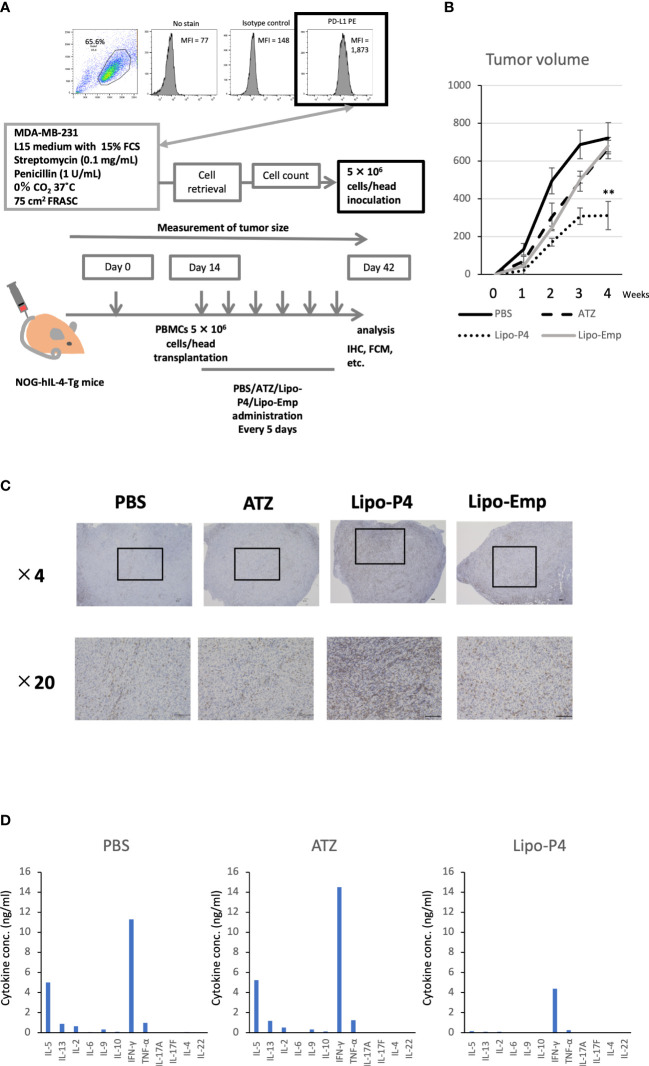
Effect of Lipo-anti-PD-L1-P4 on tumor growth in tumor-bearing humanized NOG-hIL-4-Tgmice. **(A)** Protocol for establishing the NOG-hIL-4-Tg mouse model. PD-L1 expression in MDA-MB-231 cells was analyzed using flow cytometry. **(B)** Kinetics of tumor growth in NOG-hIL-4-Tg mice. Tumor growth was measured from PBMC transplantation (Day 0). The mean volume ± standard error was calculated for each week. After four weeks, the mice were sacrificed. **P < 0.01 (one-way analysis of variance or Student’s *t*-test) (PBS [*n* = 11], ATZ [*n* = 9], Lipo-P4 [*n* = 7], Lipo-Emp [*n* = 3]). **(C)** Representative immunohistochemistry of CD3 expression in tumor tissues. Indicated bars in the tissue sections represent 100 μm. **(D)** Representative cytokine production patterns of CD3 T cells purified from NOG-hIL-4-Tg mouse spleens and stimulated with CD3/CD28. ATZ, atezolizumab; FCM, flow cytometry; FCS, fetal calf serum; IHC, immunohistochemistry; Lipo-anti-PD-L1-P4 (Lipo-P4), liposome-encapsulated anti-programmed death ligand 1 antibody-conjugated P4; Lipo-Emp, empty liposomes; MFI, mean fluorescence intensity; PBMC, peripheral blood mononuclear cell; PD-L1, programmed death ligand 1.

**Figure 6:**
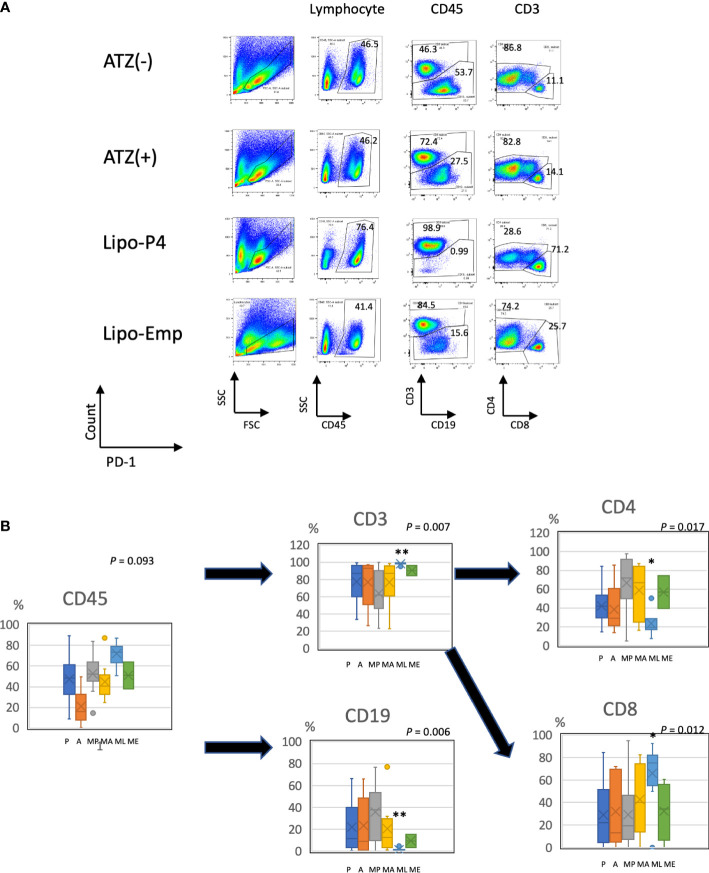
Profile of human lymphocytes in the spleen of tumor-bearing humanized mise. **(A)** Typical patterns of human lymphocytes in the NOG-hIL-4-Tg mouse spleen. Cells were gated as shown in **Supplementary Figure S1**. The gated fractions are shown above each panel. Vertical and horizontal lines represent the markers analyzed using flow cytometry. **(B)** Box-and-whisker plots of lymphocyte subsets. *P < 0.05; **P < 0.01 (one-way analysis of variance or Student’s *t*-test) (PBS [*n* = 11], ATZ [*n* = 9], Lipo-P4 [*n* = 7], Lipo-Emp [*n* = 3]). ATZ [A], atezolizumab; Lipo-Emp, empty liposome; Lipo-anti-PD-L1-P4 (Lipo-P4), liposome-encapsulated anti-programmed death ligand 1 antibody-conjugated P4; MA, MDA-MB-231 cells transplanted with atezolizumab; ME, MDA-MB-231 cells transplanted with Lipo-Emp; ML, MDA-MB-231 cells transplanted with Lipo-anti-PD-L1-P4; MP, MDA-MB-231 cells transplanted with PBS; PBS [P], phosphate-buffered saline.

To evaluate the role of immune checkpoint antibodies in immunity, we established a tumor-bearing humanized mouse model using NOG-hIL-4-Tg mice transplanted with human PBMCs (**Figure 5A**). MDA-MB-231 cells were subcutaneously transplanted into the NOG-hIL-4-Tg mice. After two weeks of observation, the mice were transplanted with PBMCs from healthy donors. Atezolizumab was administered to a subset of tumor-bearing humanized mice. The tumors engrafted and expanded (**Figure 5B**). T cell infiltration was not commonly observed in control mice (**Figure 5C**). Along with tumor growth, the percentage of B cells also increased (**Figures 6A, B**, **Table 1**). Most B cells developed into plasmablast-like cells (**Figure S3**). Tumor volume did not differ significantly between the atezolizumab-treated and control groups. However, atezolizumab-treated mice showed suppressed tumor growth and increased T cell populations, especially CD8+ T cells. Tumor-infiltrating T cells were observed in the atezolizumab-treated group (**Figure 5C**). We checked the CD45RA and CD62L memory markers and found that almost all cells were central and effector memory cells, and no significant differences were observed between the two treatment groups (**Figure S4**). These results suggest that our novel tumor-bearing humanized mouse model partially mimics breast cancer immunity, supported by our previous report on the percentage of B cells being high in patients with breast cancer (30).

**Table 1:** Proportion of human lymphocyte subsets in the tumor-bearing humanized mouse.

				/CD45		/CD3		/CD4/CD3				/CD8/CD3			
Mouse	Treatment	Spleen cell #	CD45 %	CD3 %	CD19 %	CD4 %	CD8 %	DN %	CD25 %	PD-1 %	DP %	DN %	CD25 %	PD-1 %	DP %
1	PBS	7.1E+07	54.5	29.5	70.5	92.9	7.1	17.8	7.3	33.9	41.1	57.2	31.8	5.46	5.6
2	PBS	9.8E+07	63.9	62	37.9	50.0	49.9	15.8	13.8	48.3	22.1	32	49.7	3.59	14.8
3	PBS	1.7E+07	4.98	41.4	58.6	86.5	12.4	59.7	11.6	20.5	8.19	92.5	2.5	2.5	2.5
4	PBS	4.2E+07	51.2	23.4	76.6	92.0	8.0	51	3.54	34.3	11.2	77.3	4.94	10.6	7.08
5	PBS	2.7E+07	72.7	89.5	10.7	16.4	73.1	77.6	11.9	7.63	2.84	91	5.31	2.43	1.3
6	PBS	7.8E+07	45.6	46.3	53.7	86.8	11.1	38.6	7.45	40.2	13.7	86.5	5.99	6.56	0.93
7	PBS	1.7E+08	83.9	99.7	0.22	4.93	94.9	62.6	10.9	18.3	8.16	70.4	4.31	19.5	5.76
8	PBS	8.2E+07	52.1	94	4.9	62.7	36.6	27	3.67	47.7	21.7	67.4	5.38	21.6	5.59
9	PBS	7.4E+07	35.7	53.5	45.5	97.8	1.71	16.8	6.12	51	26	49	3.41	35.5	12
10	PBS	7.8E+06	14.6	52.1	47.9	91	9	82.4	6.34	7.87	1.42	89.9	3.83	4.84	1.43
11	PBS	1.0E+08	52.2	90.2	9.78	72.1	27.8	68.1	6.27	18.9	6.7	82.9	11	4.2	1.92
**AVERAGE**	**PBS**	**7.0E+07**	**48.3**	**62.0**	**37.8**	**68.5**	**30.1**	**47.0**	**8.1**	**29.9**	**14.8**	**72.4**	**11.7**	**10.6**	**5.4**
12	ATZ	2.4E+08	87.2	92.5	5.54	26.3	73.6	18.2	16.8	42.2	22.8	56.1	34	2.95	6.91
13	ATZ	2.6E+07	24.7	87.2	12.8	87.2	66.5	27.5	12.1	47.2	13.2	27.5	12.1	47.2	13.2
14	ATZ	6.6E+07	35.9	22.9	77.1	86.2	13.7	45.2	6.04	34	14.8	70.2	8.69	16.8	4.3
15	ATZ	3.4E+07	38.5	98.6	1.4	16.2	82.4	51.5	21.4	14.2	12.9	79.7	8.21	7.68	4.36
16	ATZ	3.4E+07	40.4	98.6	1.37	23.3	76.6	59.4	24.9	8.18	7.53	69.8	10.8	12.2	7.19
17	ATZ	8.4E+07	45.8	72.4	27.5	82.8	14.1	46	9.69	31.6	12.7	60.3	19.5	13.7	6.59
18	ATZ	1.5E+07	5.3	95.6	2.89	49.5	48.7	39.7	6.03	34.5	19.7	74.6	3.08	20.4	1.91
19	ATZ	2.3E+07	29.8	68.5	32	80.4	19.5	58.5	15.9	16.8	8.74	77.5	8.61	11.1	2.85
20	ATZ	7.5E+07	57	92	7.98	66.8	33.1	73.4	7.04	15.6	3.95	75	10.5	11.3	3.17
**AVERAGE**	**ATZ**	**6.6E+07**	**40.5**	**80.9**	**18.7**	**57.6**	**47.6**	**46.6**	**13.3**	**27.1**	**12.9**	**65.6**	**12.8**	**15.9**	**5.6**
21	Lipo-P4	5.0E+04	42.1	96.7	2.9	40.6	59.1	80.9	13.9	2.63	2.59	80.9	13.9	2.63	2.59
22	Lipo-P4	2.4E+07	78.2	99.2	0.74	7.48	92.5	60.7	26.6	6.53	5.99	93.8	3.87	1.59	0.78
23	Lipo-P4	2.4E+07	79	98.9	0.99	28.6	71.2	51	4.69	37.2	7.14	86	2.67	10.5	0.81
24	Lipo-P4	1.2E+08	73.4	98.1	1.7	19.7	75.1	56	1.84	34.5	7.66	87.7	2.15	8.98	1.21
25	Lipo-P4	5.2E+07	63.1	99.5	0.17	16.9	82.1	18.3	3.07	51.5	27.1	66.2	1.51	30	2.33
26	Lipo-P4	1.4E+08	87.1	99.1	0.7	17.7	81.7	52.3	5.43	30.7	11.6	78.1	5.25	13.3	3.33
27	Lipo-P4	1.7E+07	41.6	79.3	21.2	73.8	26.1	77.7	10.2	8.31	3.74	87.4	7.91	3.73	0.91
28	Lipo-P4	1.5E+08	50.7	95.1	4.85	50.2	49.8	74.2	6.88	12.9	6	78.1	10.9	7.45	3.55
**AVERAGE**	**Lipo-P4**	**7.6E+07**	**67.6**	**95.6**	**4.3**	**30.6**	**68.4**	**55.7**	**8.4**	**25.9**	**9.9**	**82.5**	**4.9**	**10.8**	**1.8**
29	Lipo-Emp	1.7E+07	7.21	86.5	13	87.5	12.4	55.2	25.2	12.3	7.27	77.5	15.4	5.19	1.86
30	Lipo-Emp	8.8E+07	38.1	84.5	15.6	74.2	25.7	64.1	7.74	21.2	6.99	74.6	12.6	10.1	2.74
31	Lipo-Emp	1.4E+08	64	96.4	3.52	39.3	60.6	71.5	7.17	16.5	4.85	79.2	8.53	9.32	2.96
**AVERAGE**	**Lipo-Emp**	**8.1E+07**	**36.4**	**89.1**	**10.7**	**67.0**	**32.9**	**63.6**	**13.4**	**16.7**	**6.4**	**77.1**	**12.2**	**8.2**	**2.5**
32	ATZ (Low)	1.6E+07	0.18	91.2	3.04	85.6	6.48	52.4	20.4	5.11	22.1	75.7	10.8	8.11	5.41
33	ATZ (Low)	7.8E+07	53.1	76.2	22.1	96.2	2.33	41.6	2.92	43.8	11.6	74.9	11.5	9.23	4.41
34	ATZ (Low)	7.0E+07	66	99.5	0.2	20.8	78.8	17.6	1.49	47.7	33.2	73.4	1.36	21.9	3.29
35	ATZ (Low)	5.6E+07	67.7	98.7	1.07	58.5	41	36.7	15.1	30	18.2	75.3	4.96	16.2	3.52
**AVERAGE**	**ATZ (Low)**	**5.5E+07**	**46.7**	**91.4**	**6.6**	**65.3**	**32.2**	**37.1**	**10.0**	**31.7**	**21.3**	**74.8**	**7.2**	**13.9**	**4.2**
36	Lipo-P4 (Low)	9.6E+07	44.9	93	1.91	82.6	17.3	50.9	7.82	24.7	16.6	47.1	22	16.1	14.8
37	Lipo-P4 (Low)	7.4E+07	62.6	63.3	33.9	88.1	10.1	25	3.09	55.8	16.1	37.5	12	36.6	13.9
38	Lipo-P4 (Low)	4.3E+07	81.3	96.4	2.82	23.7	75.6	17.3	2.83	57.6	22.3	48.4	1.92	45.8	3.86
**AVERAGE**	**Lipo-P4 (Low)**	**7.1E+07**	**62.9**	**84.2**	**12.9**	**64.8**	**34.3**	**31.1**	**4.6**	**46.0**	**18.3**	**44.3**	**12.0**	**32.8**	**10.9**

The bold values mean that they are average of the groups shown in the upper site.

### 3.5 Lipo-anti-PD-L1-P4 suppresses the growth of PD-L1-expressing tumor cells

Next, we used our novel tumor-bearing humanized mouse model to examine the effects of Lipo-anti-PD-L1-P4 on the tumor size and immunity. We also injected empty liposomes into tumor-bearing humanized mice to determine whether tumor growth was suppressed because liposomes themselves may have an adjuvant effect. Tumor size did not differ significantly among the control (PBS), atezolizumab-treated, Lipo-anti-PD-L1-P4-treated, and empty liposome-treated groups in the first week. Thereafter, tumor growth was suppressed in the Lipo-anti-PD-L1-P4-treated group compared with that in the other groups. After four weeks, the tumor size in the Lipo-anti-PD-L1-P4-treated group was significantly smaller than that in the other three groups. The empty liposome group showed similar kinetics as the atezolizumab-treated group, suggesting that the anticancer effect of empty liposomes was induced mainly by atezolizumab. In the atezolizumab and empty liposome groups, tumor growth was initially suppressed; however, after four weeks, the tumor size was the same as that in the control group (**Figure 5B**).

We also examined the T/B and CD4+/CD8+ T cell ratios in human CD45+ cells localized in the spleen. Almost all human lymphocytes engrafted in NOG-hIL-4-Tg mice differentiated into T cells after Lipo-anti-PD-L1-P4 treatment (**Figures 6A, B**, **Table 1**). Moreover, the percentage of CD19+ B cells significantly decreased, and that of CD8+ T cells significantly increased in the Lipo-anti-PD-L1-P4-treated group. Most of the T cells identified in these groups were central and effector memory T cells and no significant difference was observed in the expression of memory markers compared to the PBS or atezolizumab-treated groups (**Figure S4**). The proportions of B and CD8+/CD4+ T cells in the empty liposome group diverged, and on average, did not differ significantly from those in the atezolizumab-treated and control groups (**Figures 6A, B**, **Table 1**). Immunohistochemistry showed that among the three (PBS, ATZ and Lipo-anti-PD-L1-P4) groups, the infiltration of T cells into the tumor was the strongest in the Lipo-anti-PD-L1-P4-treated mice (**Figure 5C**). The infiltrated cells contained CD4 T cells and CD8 T cells and expressed a significant amount of IFN-γ (**Figure S5**). We purified and stimulated these CD3+ T cells and examined whether they were functional and secreted enough cytokines (**Figure 5D**). T cells from all three treatment groups secreted a significant amount of IFN- γ and low level of TNF- α. PBS-treated T cells and atezolizumab-treated T cells also secreted IL-5 and IL-13, which are Th2 cytokines. When the CD4 and CD8 T cells were purified and independently analyzed, these cytokines were detected in CD4 T cells, and CD8 T cells secreted only IFN-γ, TNF- α and IL-2, suggesting that the CD8 T cells showed typical cytokine profiles and CD4 T cells secreted only Th1 and Th2 cytokines (**Figure S6**). Furthermore, the amount of IFN-γ secreted by Lipo-P4-aPDL1-treated mouse T cells tended to be low compared to those secreted by the cells of PBS or atezolizumab-treated mice (**Figures 5D**, **S6**). While a significant amount of IFN-γ and TNF-α was secreted by CD4 T cells of the Lipo-anti-PD-L1-P4-treated #22 mouse, no detectable cytokine production was observed for the CD8 T cells of the same mouse.

When the same reduced dose as that used clinically was administered, the anticancer effect was still observed but at a significantly lower level compared with that observed at the higher dose administration (**Figure S7**). One of the three low-dose Lipo-anti-PD-L1-P4-treated mice had more B cells and another mouse had more CD4+ T cells than CD8+ T cells (**Table 1**). These results suggested that atezolizumab, but not liposomes, slightly affected tumor growth. Lipo-anti-PD-L1-P4 showed a greater dose-dependent anticancer effect than atezolizumab in a novel tumor-bearing humanized mouse model.

### 3.6 Lipo-anti-PD-L1-P4 modulates immune cell migration in non-tumor tissues in tumor-bearing humanized mice

Lipo-anti-PD-L1-P4 is an effective anticancer agent because it suppresses tumor growth and may enhance T-cell infiltration in peripheral tissues, causing severe immune-related adverse events. Therefore, we analyzed T cell infiltration in the lungs and livers of Lipo-anti-PD-L1-P4-treated mice. T cell infiltration was more pronounced in the lungs and livers of Lipo-anti-PD-L1-P4-treated mice than in the control mice. However, tissue destruction was not severe (**Figure S8**). CD8+ T cells localized in the spleen did not express high levels of CD25 and PD-1, suggesting that cytotoxic T cell activation was moderate in the non-tumor tissues of Lipo-anti-PD-L1-P4-treated mice (**Figure S9**), while the memory phenotype of T cells was not significantly different among PBS, ATZ, and Lipo-anti-PDL1-P4 treatments (**Figure S4**).

The engraftment of human hematopoietic stem cells in immunodeficient mice is usually evaluated using CD45+ bone marrow cells. Evaluating the engraftment of human PBMCs in immunodeficient mice is difficult because CD45+ bone marrow cells cause severe graft-versus-host disease. Therefore, the T cell population in the bone marrow can help predict the adverse effects of anticancer drugs. We compared human lymphocytes in the spleen and bone marrow and calculated the correlation coefficient. In non-tumor-bearing NOG-hIL-4-Tg mice, spleen cells were positively correlated with bone marrow cells (**Figure S10**).

**Figure 7:**
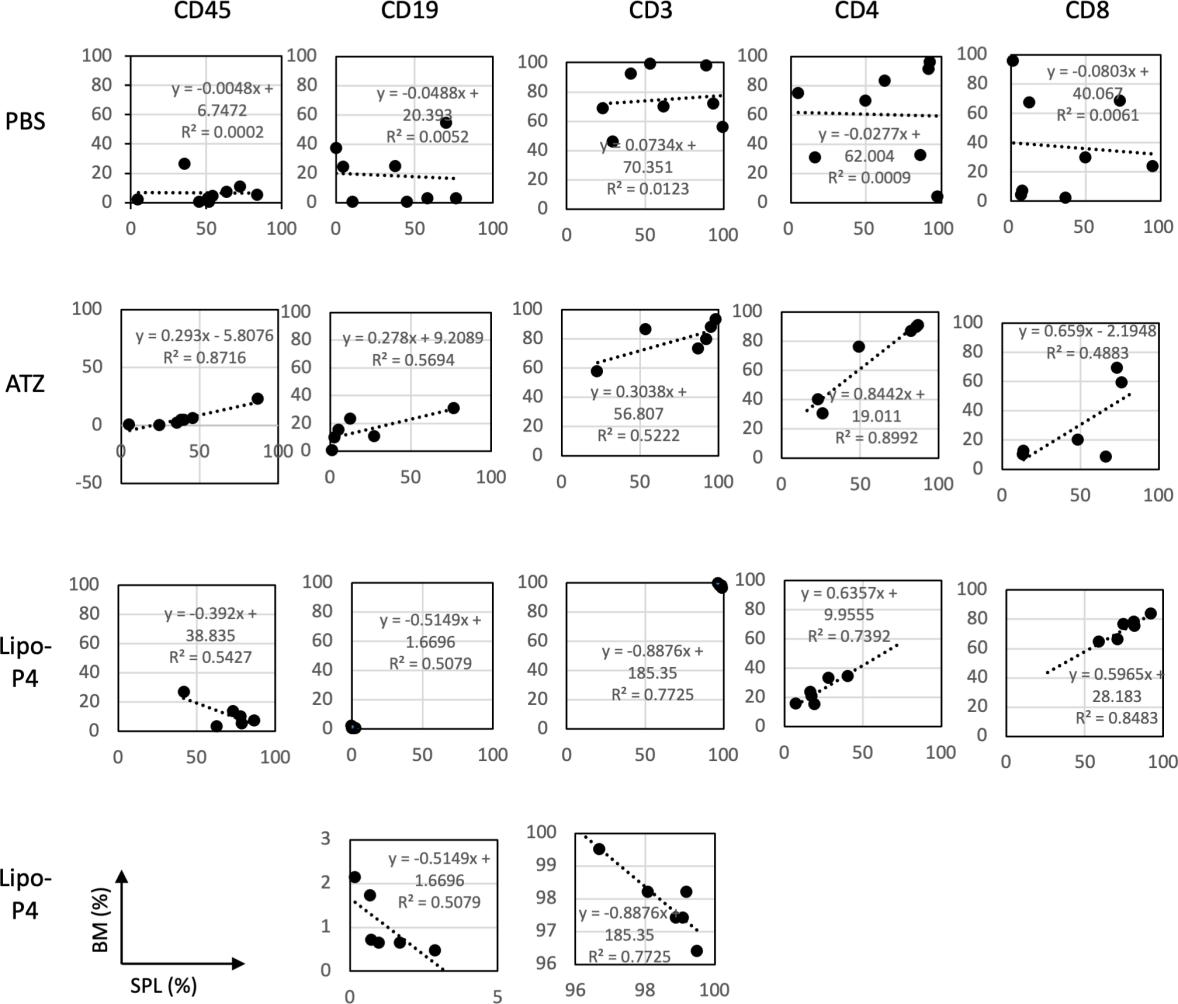
Profiles of spleen and bone marrow lymphocytes of tumor-bearing humanized NOG-hIL-4-Tg mice. The proportions (%) of human CD45+ cells/lymphocyte gated-cells, CD19+/CD45+ cells, CD3+/CD45+ cells, CD4+/CD3+ cells, and CD8+/CD3+ cells in the spleen (horizontal lines) and bone marrow (vertical lines) of NOG-hIL-4-Tg mice are plotted. Approximate straight lines and correlation coefficients are shown (PBS [*n* = 8], ATZ [*n* = 6], Lipo-P4 [*n* = 6]). ATZ, atezolizumab; Lipo-P4, liposome-encapsulated anti-programmed death ligand 1 antibody-conjugated P4; PBS, phosphate-buffered saline. Lower two panels of Lipo-P4 present the same graphs shown in the upper panels, but the vertical axis was expanded. Correlation lines and correlation coefficients (R^2^) are shown in the panels.

Next, we compared the profiles of spleen and bone marrow lymphocytes of tumor-bearing humanized NOG-hIL-4-Tg mice. There was no correlation between the proportions of lymphocyte subsets in the spleen and bone marrow of the control (PBS) mice. Conversely, atezolizumab treatment induced a positive correlation between the proportion of lymphocyte subsets in the spleen and the bone marrow of conventional NOG-hIL-4-Tg mice. The effect of Lipo-anti-PD-L1-P4 was negatively correlated with CD45+, T, and B cell proportions in the spleen and bone marrow, whereas the CD4+/CD8+ ratio was highly correlated with the proportions of the two cell types (**Figure 7**). These results suggested that the inflammation caused in non-tumor tissues by Lipo-anti-PD-L1-P4 was moderate and not severe in our tumor-bearing humanized mouse model. Spleen and bone marrow cells are complementary and not related to engraftment, in contrast to that in conventional NOG-hIL-4-Tg mice administered with atezolizumab. Lipo-anti-PD-L1-P4 treatment increases the efficiency of human lymphocyte engraftment into peripheral lymphoid tissues. Meanwhile, severe inflammation in the peripheral non-tumor tissues was avoided.

## 4 Discussion

In this study, we showed that Lipo-anti-PD-L1-P4 suppressed tumor growth without causing severe inflammation in the peripheral tissues of tumor-bearing humanized NOG-hIL-4-Tg mice. The effect was 10 times higher than that of free P4 in terms of tumor cell growth, and CD8+ T cell proliferation was considered.

We recently reported that the high-dose of P4 treatment induced transient suppression of T cell activation and enhancement of T cell localization in the spleen (18), whereas previous studies demonstrated the anticancer effects of P4 (13, 14). As MDA-MB-231 cells express the membrane P4 receptor PGRMC1 (31), and P4 suppresses cell proliferation by inhibiting PGRMC1, the anticancer effect was predicted. However, to the best of our knowledge no *in vivo* studies have reported tumor growth suppression or non-tumor tissue protection by P4.

Our results suggest that P4-based immunoregulatory agents are immuno-protective and possess antitumor effects, demonstrating the potential of immunoregulatory drugs for cancer treatment. Therefore, these agents may be safer than cortisol, which diminishes the immune response. However, the concentration required to effectively suppress the immune system and tumor growth was approximately 200 μM, which is 10-fold higher than its physiological concentration. This observation highlights the difficulty in using P4 as an alternative immune regulator to cortisol. Therefore, we aimed to deliver P4 to cells more efficiently.

Previously, it was believed that steroid hormones were secreted via simple diffusion (32). However, vesicle-mediated steroid hormone secretion has recently been reported in *Drosophila* (33). Syncytiotrophoblasts secrete large amounts of P4 (11) and exosomes into the placental villi (21). We hypothesized that exosomes would support the delivery of steroids. By encapsulating P4 in liposomes, the suppression of T cell activation and cancer cell proliferation was induced in the presence of physiological concentrations of P4 *in vitro*. In the tumor-bearing humanized mouse model, while tumor growth was suppressed and the proportion of CD8+ T cells increased, T cell infiltration into peripheral tissues did not significantly increase, and no symptoms of graft-versus-host disease were observed. Empty liposomes did not suppress tumor growth in the same manner as Lipo-anti-PD-L1-P4 did. Moreover, the empty liposomes did not enhance CD8+ T cell expansion in the spleen. These results suggested that the anticancer effect was induced by P4. A similar phenomenon may occur at the maternal–fetal interface to suppress anti-fetal immunity. Therefore, the effect of Lipo-anti-PD-L1-P4 may mimic the regulation of extravillous trophoblast migration and/or survival, and allo-specific immune activation.

During pregnancy, women develop an immune tolerance, which allows them to accept a semi-allogeneic fetus (34). In the mother, placental trophoblasts, which share the same characteristics as cancer cells, are confined to the uterus (6, 7). P4 has multiple functions involved in the trophoblast confinement (11). P4 has been reported to induce IL-4 secretion and Th2 shift, which do not enhance cytotoxicity, but enhance B-cell responses (12, 35). We previously reported that the proportion of CD8+ T cells was significantly increased in P4-pretreated PBMC-transplanted NOG-hIL-4-Tg mice (18). Kawata et al. (36) reported that short-term exposure of T cells to high concentrations of P4 induced the transient expression of T-bet. Therefore, a high concentration of P4 may induce transient T-bet expression in placental T cells to acquire the Th1 phenotype. During pregnancy, serum glucocorticoid levels increase because of stress (37). While glucocorticoids irreversibly diminish the immune system, increased levels of P4 may suppress glucocorticoid function to protect the mother’s immune system from T cell exhaustion. P4 suppresses glucocorticoid function, which may suppress the development of B-cell-derived plasmablasts, resulting in the absence of B cells in the humanized mouse model.

Humanized mouse models are a powerful tool for evaluating immune-related drugs because the immune system is highly divergent between species. Many platforms have been constructed to analyze the human immune microenvironment (38, 39). However, to date, no such system has been used to evaluate mature B cell responses (26). Various platforms have been constructed to evaluate the effects of antibody-based anticancer drugs on human immunity in immunodeficient mice (40). However, these models are based on T cells and the innate immune response. The humanized mouse model used in this study was established using NOG-hu-IL-4-Tg mice transplanted with human PBMCs (27). Human B cells engraft, and a large proportion differentiate into plasmablast-like cells. Interestingly, only the transplantation of cancer cell lines tended to increase B cell subsets in our humanized mouse model. Xu et al. (41) reported similar B-cell subsets in their wild-type mouse model. The cancer microenvironment may induce glucocorticoid production because glucocorticoids are stress hormones. Glucocorticoids have been reported to enhance BLIMP-1 expression (42), which may promote premature B-cell differentiation into autoreactive plasmablasts. Recently, B-reg, an IL-10-secreting B-cell subset, was reported to have a phenotype similar to that of plasmablasts (43). Thus, it is plausible that B cells contribute to the cancer microenvironment. B cells have been previously reported to promote tumor growth by producing IL-10 (44). An increase in plasmablasts may create an environment that promotes cancer immune evasion, whereas the presence of B cells in the tumor microenvironment is reported to be associated with anticancer immunity if memory B cells are dominant (45). Garaud et al. (46) reported that plasmablast-like B cells may become immunosuppressive and promote tumor growth. In humanized mice, B cells may promote tumor growth in a similar manner. We previously reported that patient-derived plasmablasts secrete antibodies but produce specific antibodies at low levels (28). The cytokines secreted by B cells may be immunosuppressive. Non-specific or autoreactive antibodies may interact with Fc receptors to block optical surveillance of opsonized cancer cells by natural killer cells and macrophages. This mouse model may be sufficient to elucidate the functions of B cells in the cancer microenvironment.

Lipo-anti-PD-L1-P4 induces T cell infiltration in cancer tissues and decreases plasma cell levels. However, the underlying mechanism is unclear, although one study showed a reduction in the number of plasmablasts in P4 cultures (47). Here, in PBMCs from healthy donors, almost all the B cells were naïve memory/transitional B cells and not plasmablasts. As non-activated naïve and memory B cells may survive in a high P4 environment, similar to the T cell fate in P4, most B cells might survive in healthy donors. If the environment is replaced by a P4-induced anticancer immune environment, plasmablasts may disappear and cytotoxic T cells may become dominant.

Lipo-anti-PD-L1-P4 did not significantly enhance peripheral tissue inflammation. This was another striking feature of P4. Previously, we reported that a high concentration of P4 suppresses T-cell activation (18). Similarly, Papapavlou et al. (48) reported that P4 inhibits T cell activation at high concentrations, which may be an immune regulatory function specific to P4. We found an increase in CD8 T cell number and a decrease in IFN-γ secretion per cell, which might increase the threshold of inflammation caused by low-affinity autoreactive T cells that have migrated in the peripheral tissues. However, we need to examine the possibility that the Lipo-anti-PD-L1-P4-treated T cells extensively attacked the cancer cells and were highly exhausted after one month of action, and the underlying molecular mechanisms need to be clarified in future studies.

While the 6 h treatment with a high-dose of P4 was enough for the remodeling of the lymphocyte profile and characteristics in humanized mice (18), the P4 leakage from the liposome was rather quick under biological conditions. Therefore, although the anticancer drug may be functional in this liposomal formulation, further studies are required for its optimization and implementation. First, the stable encapsulation of the P4 in the formula is essential. As we observed a dose-dependent effect of P4, it is crucial to encapsulate quantitative amount of P4 in liposomes. The composition of liposomes should also be determined by extensive examination of the entrapment efficiency, toxicity, and stability. The scalability of the key outcome is also important for the implementation. As for the entrapment efficacy and loading percentage, electromicroscopic analyses are needed. It is also necessary to determine how Lipo-anti-PD-L1-P4 can be used in the treatment. A large portion of liposomes tend to accumulate in the liver and lung, in which capillaries are abundant. Therefore, it might be difficult to target the liposomes to the tumor. For this purpose, the ratio of antibodies and the diameter of the liposomes should be optimized. Moreover, the route of administration should be optimized. Along with these optimization studies, pharmacokinetic and biodistribution studies should be performed.

Second, as the killing efficiency of Lipo-anti-PD-L1-P4 against Karpas 707H was lower than that against MDA-MB-231 and JEG-3 cells, its cancer treatment efficacy may be limited. Further experiments should be conducted using other cell types. Especially, since progesterone is a sex steroid so its relationship with reproductive organ-related cancers such as ovarian and prostate cancer should be examined. Moreover, as we used cancer cell lines, further analyses of heterogeneous cancer tissues should be performed. Cancer screening is required to determine the limitations of mice transplanted with patient-derived xenografts.

Third, our mice did not develop lymph nodes that contained human lymphocytes. Therefore, lymph node metastasis could not be analyzed. Fourth, B cells differentiated into plasmablasts in the mouse model. Fifth, the mice used human PBMCs for the immune humanization. Therefore, the cells have a limitation of survival period. This makes it difficult to evaluate the survival rate of tumor-bearing mice after several months, because the number of human immune cells decrease after three months and the effect of lymphocytes could not be evaluated correctly. There is a need to establish a new humanized mouse model to maintain lymphocytes in a naïve/memory state and evaluate the contribution of these cells to the anticancer effect. Other issues may be addressed using our model. However, if the relationship between *in vitro* and *in vivo* (patients and humanized mice, respectively) analyses is confirmed, the mouse system will become a useful tool for evaluating the response of patients to ICIs.

Nevertheless, our results strongly suggested Lipo-anti-PD-L1-P4 as an anticancer drug candidate. Lipo-anti-PD-L1-P4 suppressed the hyperactivation of acquired immunity and exerted a strong anticancer effect. It also induced a shift in the cancer immune microenvironment towards a state of inducing tumor T cell infiltration, tumor necrosis, and natural killer/T cell proliferation, and inhibiting plasmablast-like cell differentiation. To date, no other molecule has simultaneously achieved these effects. Lipo-anti-PD-L1-P4 enhances T-cell infiltration into the tumor tissue, resulting in the suppression of tumor growth. Thus, Lipo-anti-PD-L1-P4 is a promising anticancer drug. Our tumor-bearing humanized mouse model is a promising tool for the development of immune-related anticancer drugs.

## Data availability statement

The original contributions presented in the study are included in the article/**Supplementary Material**. Further inquiries can be directed to the corresponding author.

## Ethics statement

The studies involving human participants were reviewed and approved by Tokai University Human Research Committees. The patients/participants provided their written informed consent to participate in this study. The animal study was reviewed and approved by Animal Care Committees of Tokai University School of Medicine and Central Institute for Experimental Animals.

## Author contributions

YK, RI, and YM contributed to the conception and design of the study. RI, TosS, YM, MMa, MT, and YK contributed to the development of the methodology. HK, SO, YO, TomS, SY, NK, YG, DK, KI, TM, and AY contributed to the acquisition and analysis of data. YK, RI, YM, MMi, KA, HI, and TosS contributed to the writing, review, and/or revision of the manuscript. HK, TaS, YG, RI, and BT provided administrative, technical, or material support. KF, MMi, KA, HI, TaS, and YK supervised the study. All authors contributed to the article and approved the submitted version.
