# Supplementary material for: Construction of the systemic anticancer immune environment in tumour-bearing humanized mouse by using liposome-encapsulated anti-programmed death ligand 1 antibody-conjugated progesterone
Source: Front Immunol. 2023 Jul 10;14:1173728. doi: 10.3389/fimmu.2023.1173728 (PMC10364058; doi:10.3389/fimmu.2023.1173728)

Supplementary Material

Construction of the Systemic Anticancer Immune Environment in Tumor-Bearing Humanized Mouse by Using Liposome-Encapsulated Anti-Programmed Death Ligand 1 Antibody-Conjugated Progesterone

Yoshie Kametani*, Ryoji Ito, Shino Ohshima, Yoshiyuki Manabe, Yusuke Ohno, Tomoka Shimizu, Soga Yamada, Nagi Katano, Daiki Kirigaya, Keita Ito, Takuya Matsumoto, Koichi Fukase, Banri Tsuda, Hirofumi Kashiwagi, Yumiko Goto, Atsushi Yasuda, Masatoshi Maeki, Manabu Tokeshi, Toshiro Seki, Mikio Mikami, Kiyoshi Ando, Hitoshi Ishimoto, Takashi Shiina

*** Correspondence:** Corresponding Author: Yoshie Kametani [ky49214@tsc.u-tokai.ac.jp](mailto:ky49214@tsc.u-tokai.ac.jp)


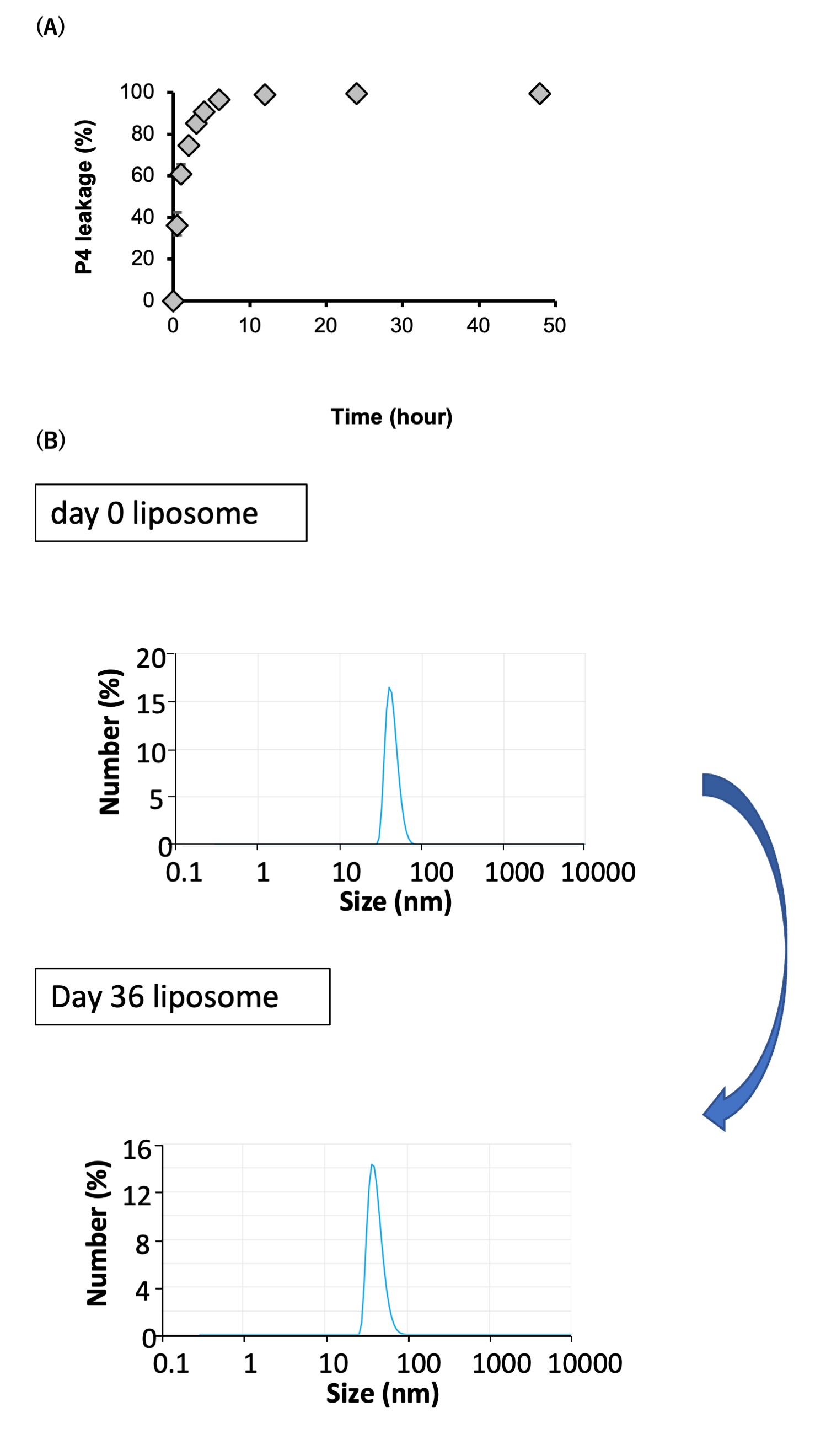


**Figure S1.** **Stability of the Lipo-anti-PD-L1-P4.**

(A) Representative result of liposome leakage test of Lipo-anti-PD-L1-P4. (B) Representative result of size distribution at day 0 and day 36 of the preparation. Lipo-anti-PD-L1-P4 was stored at 4℃. Upper panel; day 0, Lower panel; day 36.


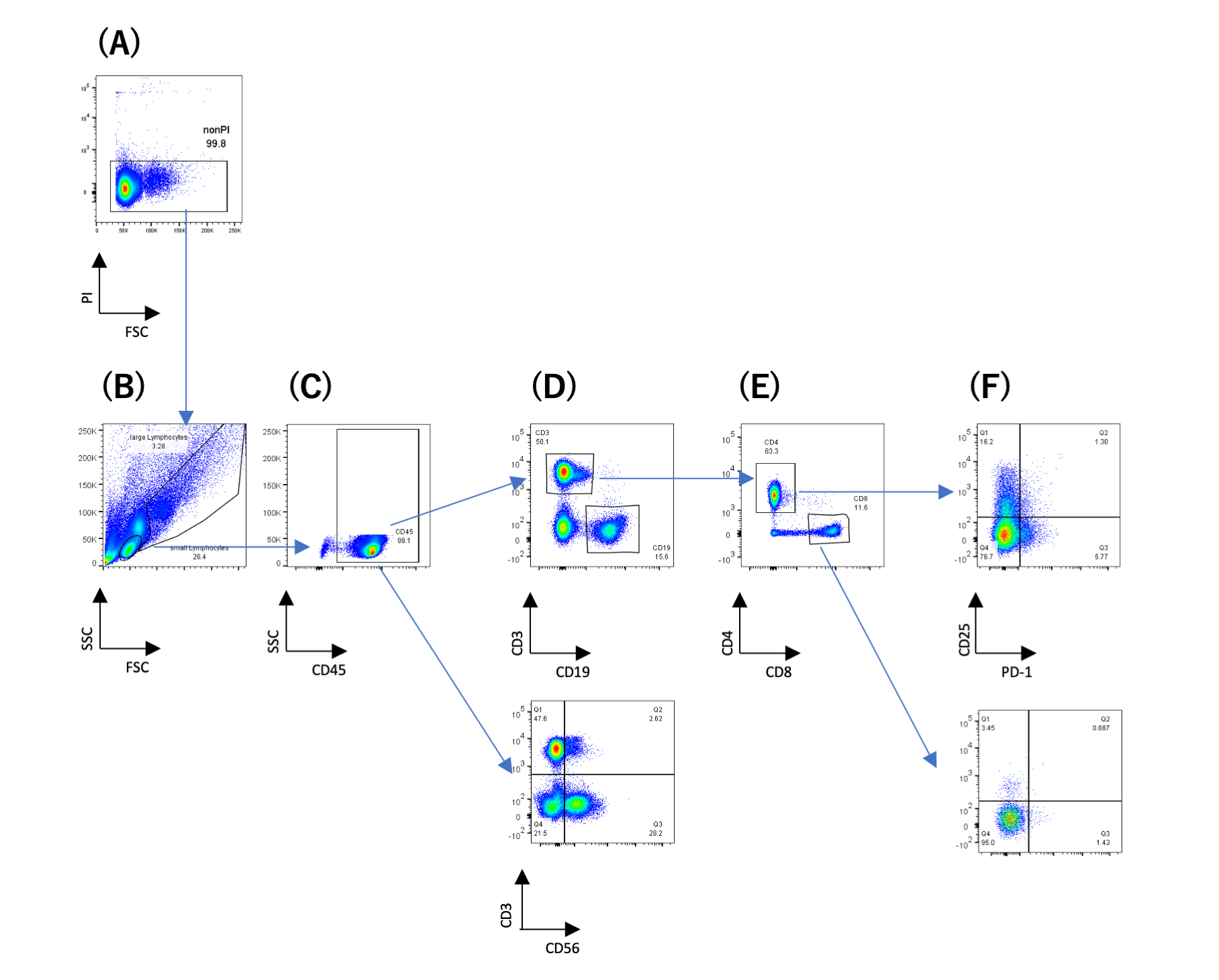


**Figure S2.** **Gating strategy of the FCM pattern of human lymphocytes.**

Living PBMCs were assessed using propidium iodide (PI)-staining (A), and the lymphoid-gated cells were selected using the forward and side scatters (FSC/SSC) parameters (B). Lymphocyte-gated cells were then gated for human CD45+ leukocytes (C). The CD45+ cells were analyzed for CD3 T, CD19 B, and CD56 NK cells (D). CD3-gated cells were further divided based on CD4 and CD8 expression (E). Each cell fraction was further analyzed for CD25 and PD-1 expression (F).

**
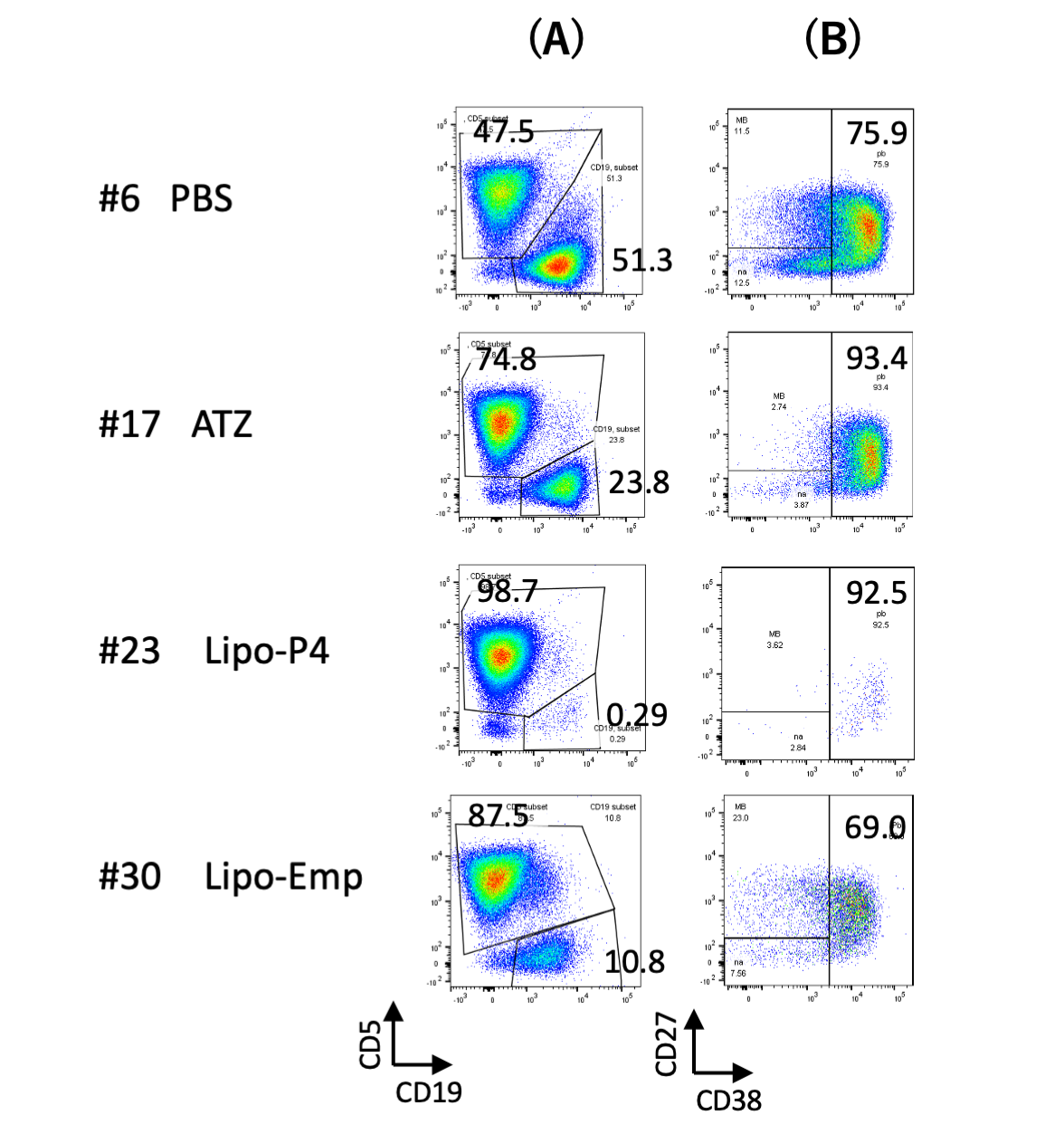
**

**Figure S3. Phenotype of the human B cells engrafted in the tumor-bearing humanized mice.**

Living human CD45+ leukocytes were gated as mentioned in Figure S1. CD19 and CD5 expressions were analyzed for these CD45+ cells, to distinguish T cells, B1, and transitional B cell detection (A). B cell ratios are less than those shown in Table 1 because CD5+ B cells were eliminated. The CD19 + cells were analyzed for CD27 and CD38 expression, for detecting plasmablasts (B). The numbers in each panel represent the proportion (%) of each fraction. The mouse number used for the panel is shown.


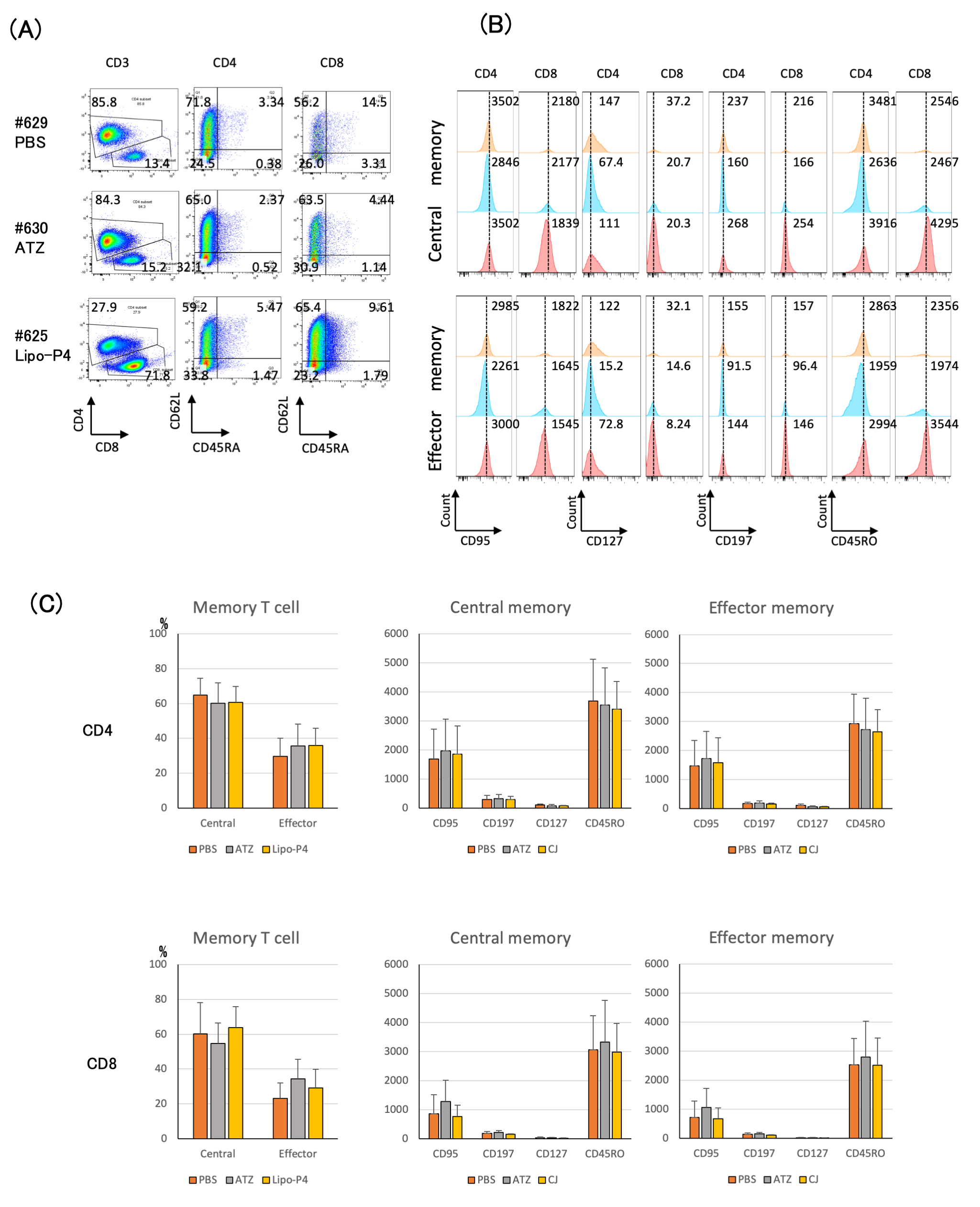


**Figure S4.** **Phenotype of the human memory T cells engrafted in the tumor-bearing humanized mice.**

Representative flow cytometry patterns of memory T cells engrafted in the tumor-bearing humanized mice are shown. Living human CD45+CD3+ leukocytes were gated as mentioned in Figure S1. CD4 and CD8 expressions were analyzed for these CD3+ cells, to distinguish Th and Tc cells. CD62L and CD45RA expressed on Th cells (CD4) and Tc cells (CD8) were further analyzed and CD62L single positive cells were defined as central memory T cells and double negative cells as effector memory T cells (A). The expressions of CD95, CD127, CD197, and CD45RO were analyzed for Th cells and Tc cells of central (CD62L SP) and effector memory (DN) T cells (B) The numbers in each panel represent the proportion (%) of each fraction for (A) or MFI for (B). (C). Representative FACS patterns of these differentiation markers are shown (B). The Percentage of central and effector memory T cells in CD4/CD8 T cells (left panels) and mean MFIs of these markers (right panels) are shown in (C).


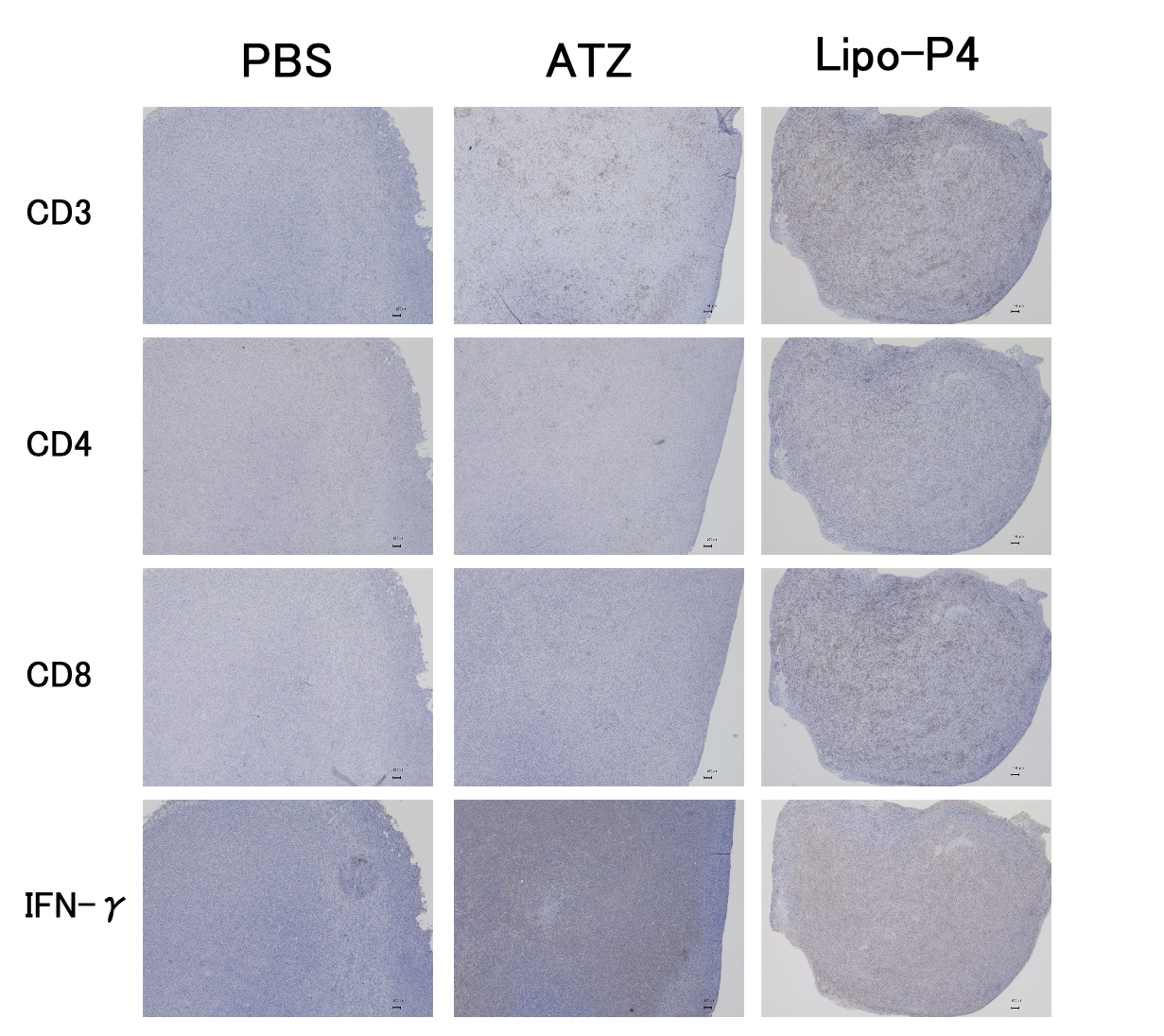


**Figure S5. Representative immunohistochemistry of the T cell phenotype infiltrated in the tumor tissues.**

CD3, CD4, CD8, and IFN-γ expression in the tumor-infiltrated human T cells were compared among the control, atezolizumab, and Lipo-anti-PD-L1-P4 treated mice. Indicated bars in the tissue sections represent 100 μm. ATZ, atezolizumab; FCM, flow cytometry; Lipo-anti-PD-L1-P4 (Lipo-P4), liposome-encapsulated anti-programmed death ligand 1 antibody-conjugated P4.


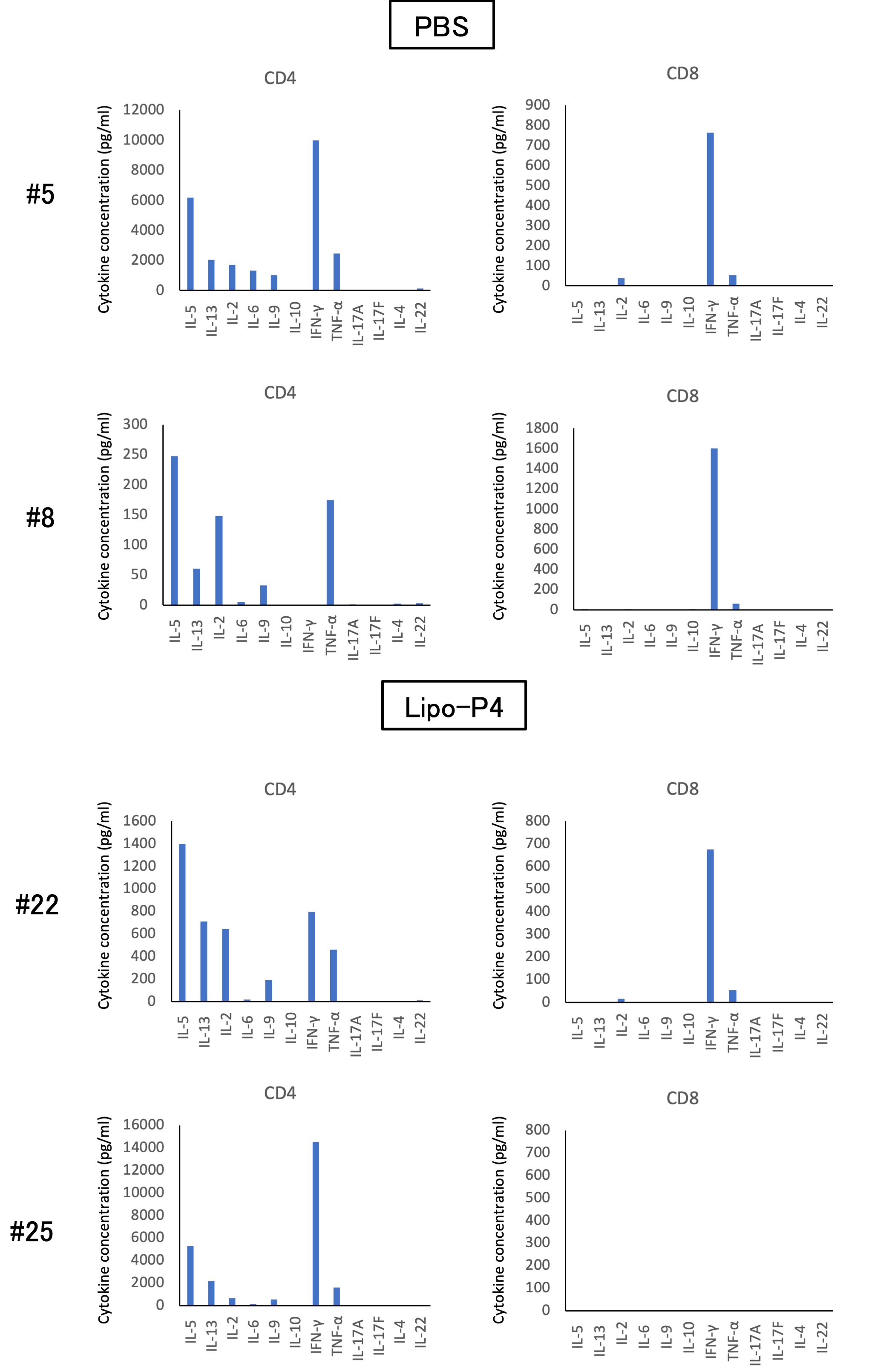


**Figure S6. Representative immunohistochemistry of the T cell phenotype infiltrated in the tumor tissues.**

CD3, CD4, CD8, and IFN-γ expression in the tumor-infiltrated human T cells were compared among the control, atezolizumab, and Lipo-anti-PD-L1-P4 treated mice. Indicated bars in the tissue sections represent 100 μm. ATZ, atezolizumab; FCM, flow cytometry; Lipo-anti-PD-L1-P4 (Lipo-P4), liposome-encapsulated anti-programmed death ligand 1 antibody-conjugated P4.

**
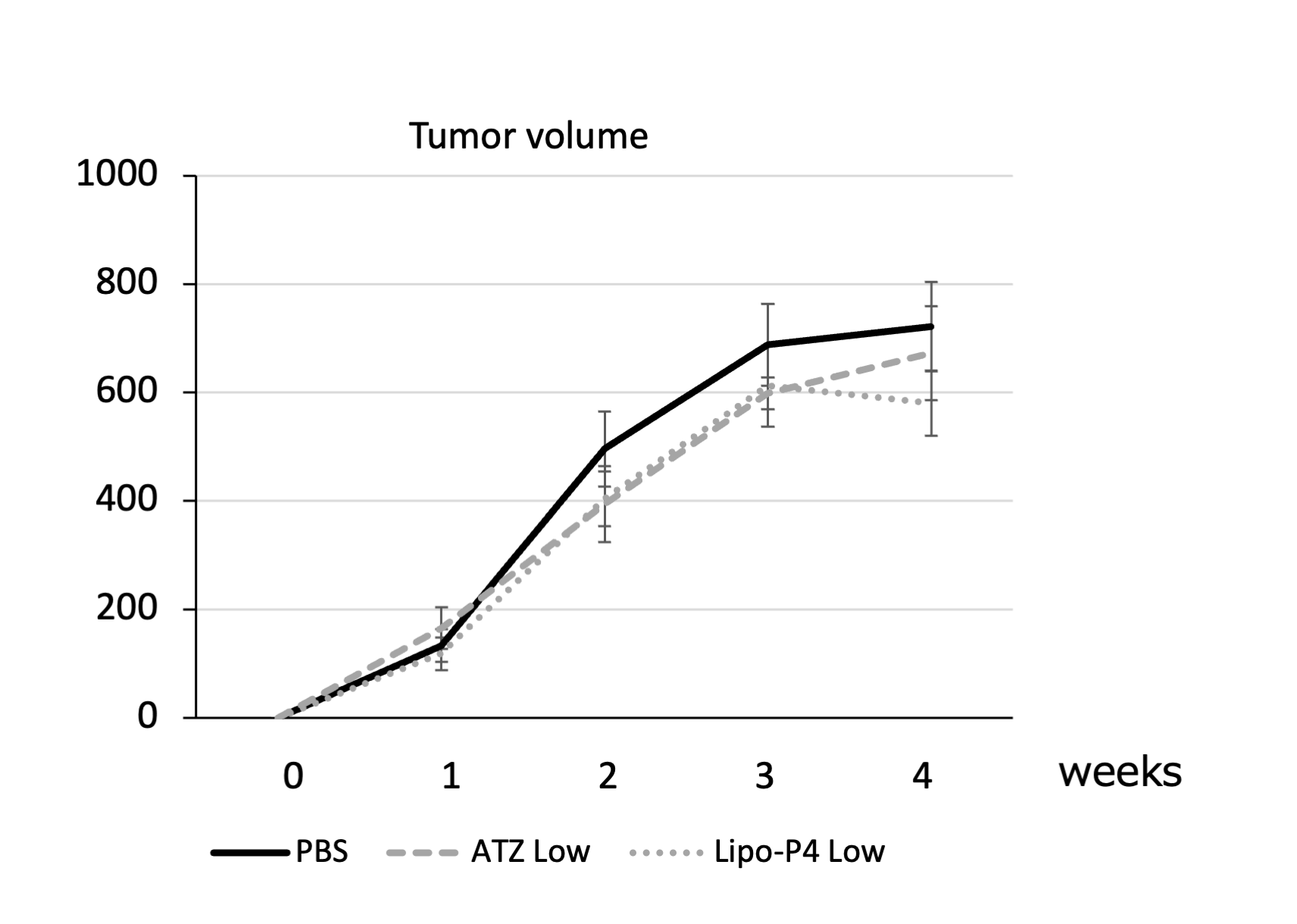
**

**Figure S7.** **The effect of Low-dose P4 administration.**

The kinetics of tumor growth of tumor-bearing PBL-NOG-hIL-4-Tg. The tumor growth was measured from the PBMC-transplantation (day 0) and the mean volume (± S.E) was calculated for each week and shown. After four weeks, mice were sacrificed and analyzed. MP; n=11, MA-low; n=4, MP-low; n=3.

**
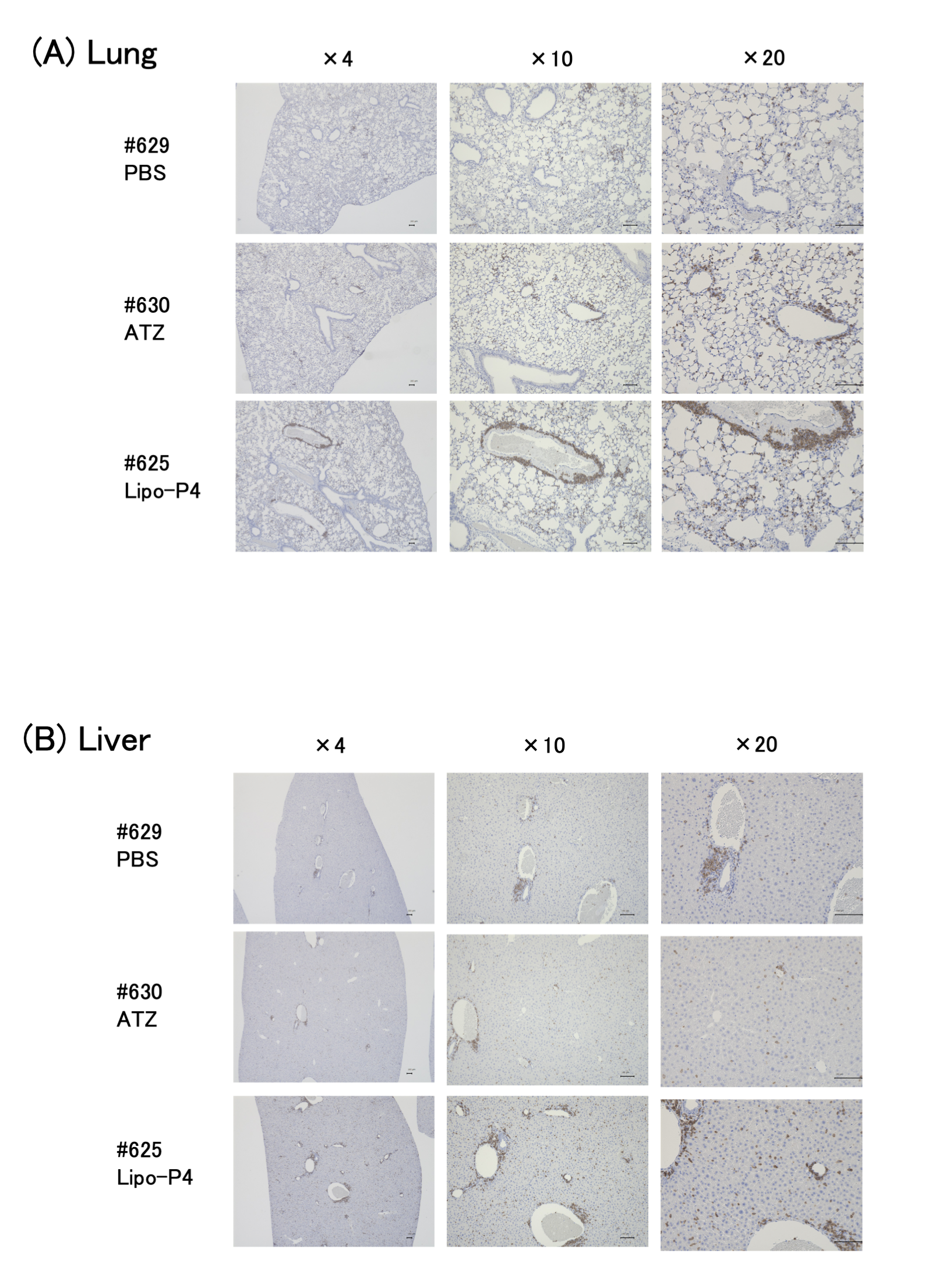
**

**Figure S8.** **T cell infiltration into the lung and liver tissues of the tumor-bearing humanized mice.**

Representative IHC patterns of peripheral tissues for CD3 positive cell infiltration into tumor-bearing PBL-NOG-hIL-4-Tg. (A) Lung sections. (B) Liver sections. Brown cells are T cells infiltrated into the tissues.


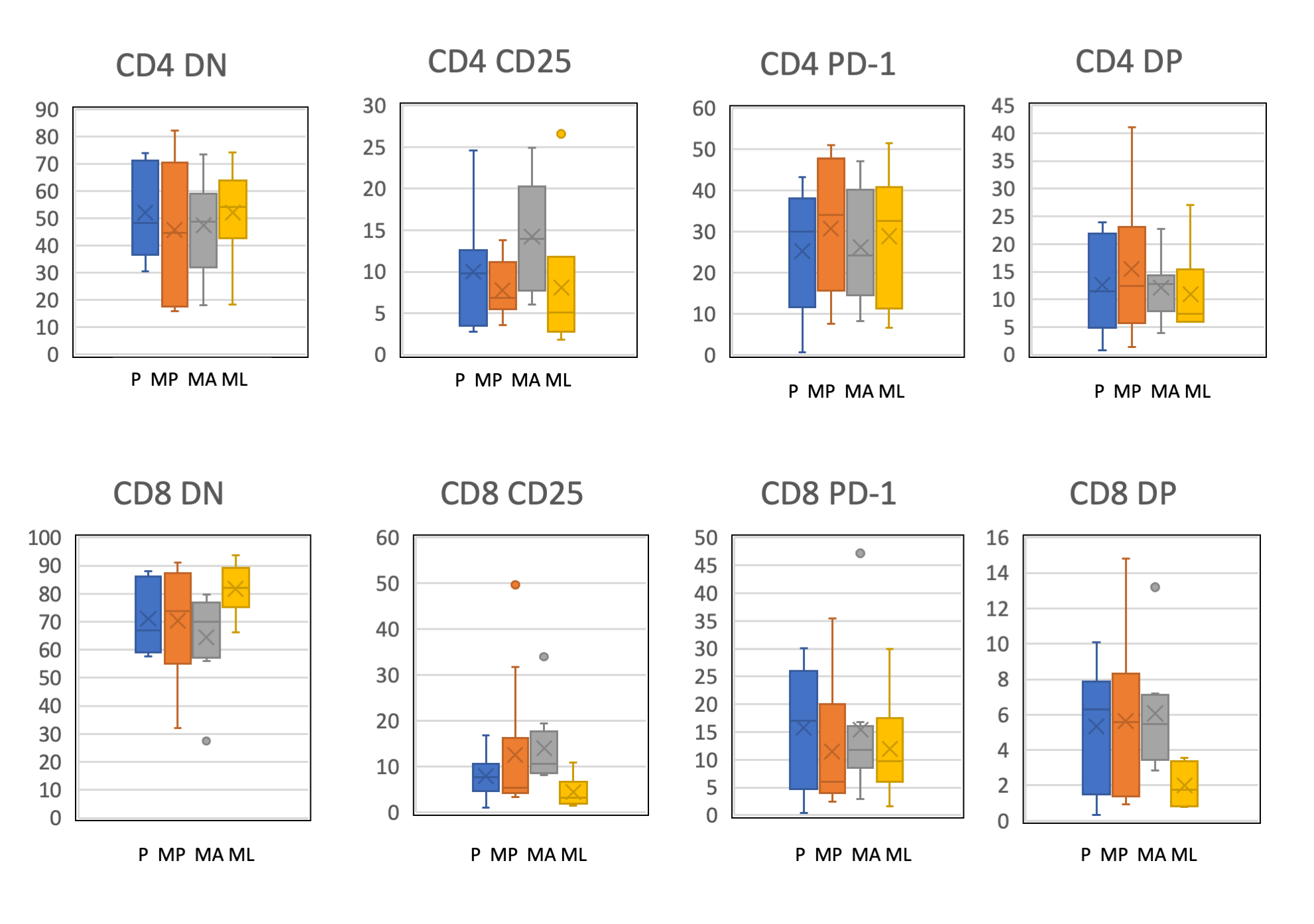


**Figure S9.** **Activation/exhaustion profile of human lymphocytes in the tumor-bearing humanized mouse spleen**.

Box-and-whisker plots of each lymphocyte subset. Non-tumor-bearing mice are also involved. Upper panels; CD4 T cells, Lower panels; CD8 T cells. P; PBS, MP; MDA-MB231-transplanted PBS, MA; MDA-MB231-transplanted atezolizumab, ML; MDA-MB231-transplanted Lipo-P4-aPDL1. M; n= 8, MP; n=10, MA; n=8, ML; n=6. (Mice with spleen cell engraftment >2 ×10^7^ were selected and analyzed further). Student’s t-test and one-way ANOVA were performed, but no significance was observed across all treatment groups.


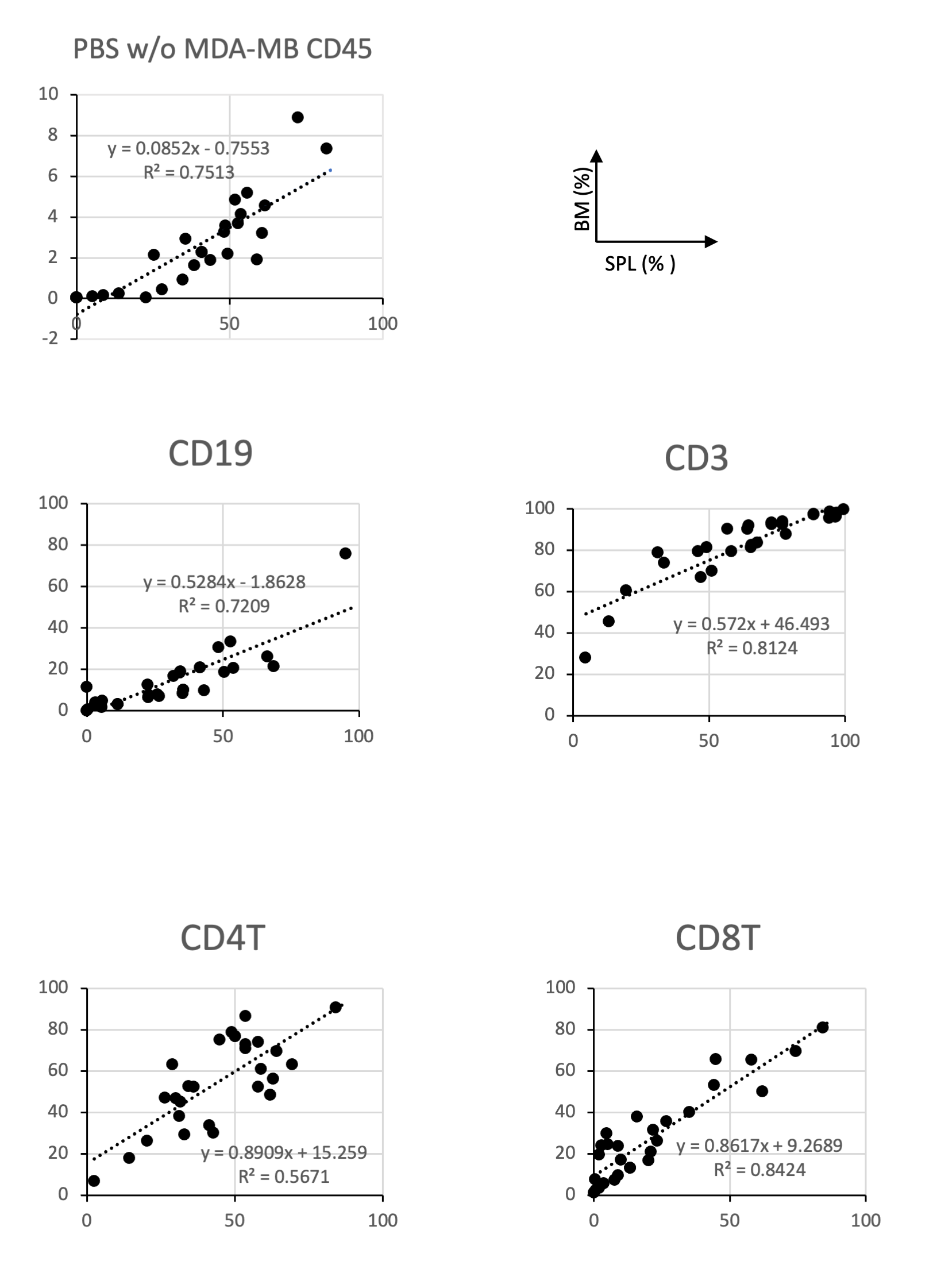


**Figure S10. Correlation of spleen and bone marrow lymphocyte proportion in PBL-NOG-hIL-4-Tg.**

The comparison of PBL-NOG-hIL-4-Tg mouse spleen cells with bone marrow cells. The proportions of human CD45+/total cells, CD19+/CD45+ cells, CD3+/CD45+ cells, CD4+/CD3 cells, CD8+/CD3 cells in the spleen and bone marrow of each mouse were plotted. Approximation straight lines and correlation coefficients are shown in each graph. (n=30)

**Supplementary Table 1. List of Antibodies used for FCM and IHC**
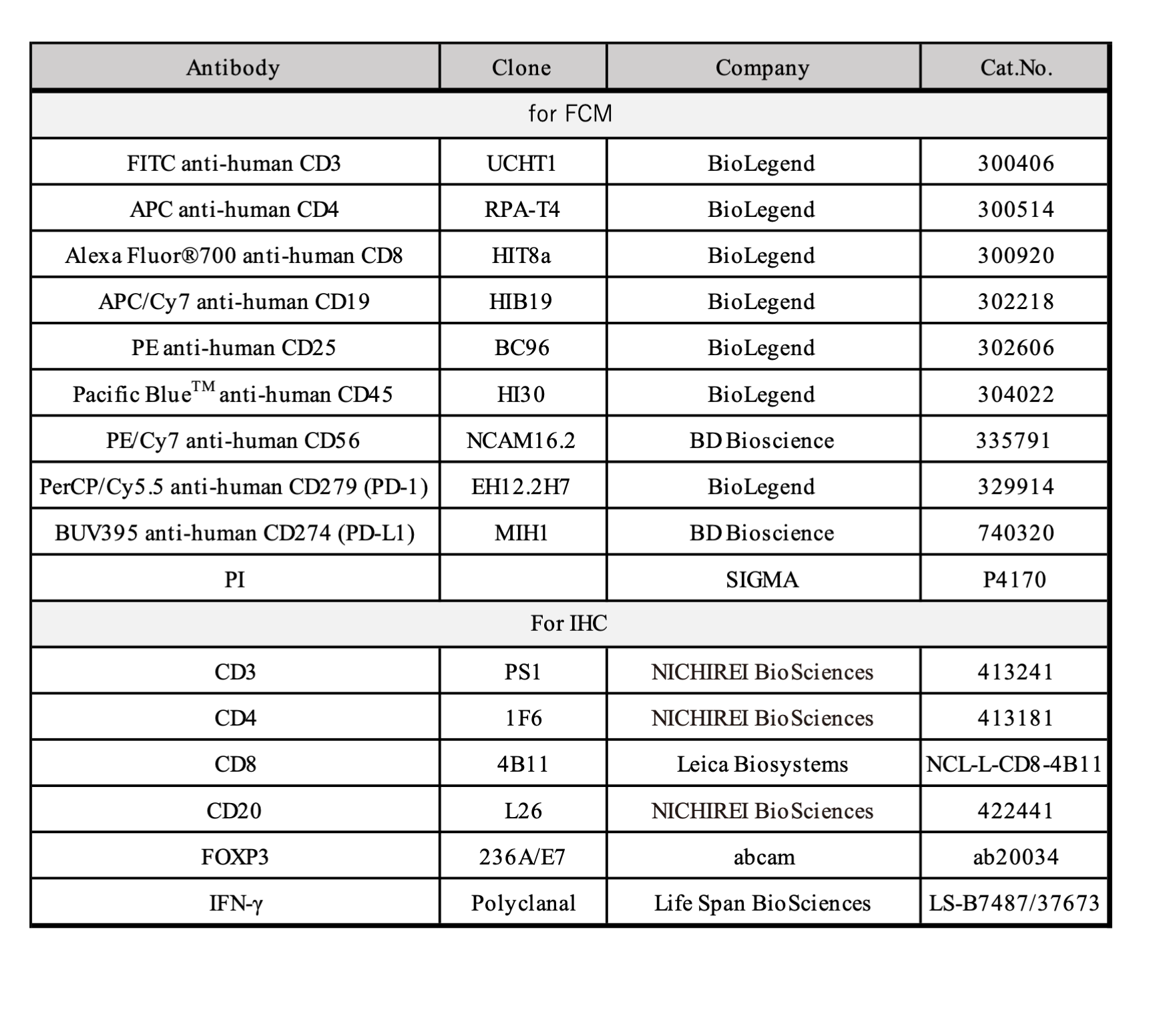

Supplement: Supplementary file 1 [file DataSheet_1.docx]
